# The association between dietary factors and gestational hypertension and pre-eclampsia: a systematic review and meta-analysis of observational studies

**DOI:** 10.1186/s12916-014-0157-7

**Published:** 2014-09-22

**Authors:** Danielle AJM Schoenaker, Sabita S Soedamah-Muthu, Gita D Mishra

**Affiliations:** School of Population Health, University of Queensland, Brisbane, Queensland Australia; Division of Human Nutrition, Wageningen University, Wageningen, The Netherlands

**Keywords:** Diet, Maternal nutrition, Meta-analysis, Pre-eclampsia, Pregnancy-induced hypertension, Systematic review

## Abstract

**Background:**

Dietary factors have been suggested to play a role in the prevention of hypertensive disorders of pregnancy (HDP), including gestational hypertension and pre-eclampsia, but inconsistent findings have been reported. A systematic review and meta-analyses were performed to synthesize evidence from observational studies of reproductive-aged women on the association between dietary factors and HDP.

**Methods:**

MEDLINE and EMBASE were searched to identify studies published until the end of May 2014. Studies were included if they were observational studies of reproductive-age women and reported results on dietary factors (energy, nutrients, foods or overall dietary patterns, alone or in combination with dietary supplements) and gestational hypertension and/or pre-eclampsia. Studies were excluded if they reported on supplements not in combination with dietary intake, or examined a biomarker of dietary intake. Random effects meta-analyses were performed on calculated weighted mean differences (WMD) of dietary intake between cases and non-cases, and effect estimates were pooled.

**Results:**

In total, 23 cohort and 15 case–control studies were identified for systematic review, of which 16 could be included in the meta-analyses. Based on meta-analyses of cohort studies, unadjusted energy intake was higher for pre-eclampsia cases (WMD 46 kcal/day, 95% confidence interval (CI) −13.80 to 106.23; *I*^2^ = 23.9%, *P =* 0.26), although this was not statistically significant. Unadjusted intakes of magnesium (WMD 8 mg/day, 95% CI −13.99 to −1.38; *I*^2^ = 0.0%, *P* = 0.41) and calcium (WMD 44 mg/day, 95% CI −84.31 to −3.62, *I*^2^ = 51.1%, *P* = 0.03) were lower for the HDP cases, compared with pregnant women without HDP. Higher calcium intake consistently showed lower odds for HDP after adjustment for confounding factors (OR = 0.76, 95% CI 0.57 to 1.01, *I*^2^ = 0.0%, *P* = 0.79). A few studies examining foods and dietary patterns suggested a beneficial effect of a diet rich in fruit and vegetables on pre-eclampsia, although not all the results were statistically significant.

**Conclusions:**

Based on a limited number of studies, higher total energy and lower magnesium and calcium intake measured during pregnancy were identified as related to HDP. Further prospective studies are required to provide an evidence base for development of preventive health strategies, particularly focusing on dietary factors during pre-pregnancy and early pregnancy.

Please see related article: http://www.biomedcentral.com/1741-7015/12/176/abstract.

**Electronic supplementary material:**

The online version of this article (doi:10.1186/s12916-014-0157-7) contains supplementary material, which is available to authorized users.

## Background

Hypertension represents the most common complication of pregnancy, affecting up to 15% of pregnancies worldwide [1,2]. Hypertensive disorders of pregnancy (HDP) include gestational hypertension, generally defined as new-onset hypertension (≥140 mmHg systolic or ≥90 mmHg diastolic blood pressure) arising after 20 weeks’ gestation, and pre-eclampsia, defined as gestational hypertension accompanied by proteinuria (excretion of ≥300 mg protein every 24 hours) [1,3]. These disorders are a major cause of maternal and perinatal morbidity and mortality [2], and result in an increased future risk for cardiovascular disease [4,5] and type 2 diabetes mellitus [4,6] for both mother and offspring. These lifelong and inter-generational adverse health consequences highlight the need for identification of preventive strategies.

Although the etiology of gestational hypertension and pre-eclampsia remains largely unclear, evidence suggests that diet may play a role. HDP are characterized by metabolic disturbances similar to those found in cardiovascular diseases and type 2 diabetes mellitus (T2DM) including endothelial dysfunction, inflammation, oxidative stress, insulin resistance and dyslipidemia [1,2]. Diet is a well-known risk factor for cardiovascular disease and T2DM [7,8]. Furthermore, serum nutrient levels (such as elevated polyunsaturated fatty acids, and decreased vitamins C and E, zinc, and iron) have been associated with increased inflammation, oxidative stress, and dyslipidemia [9,10]. Nutrient status has also been directly linked with increased risk of pre-eclampsia, including increased serum triglyceride and fatty acids, and reduced levels of serum calcium, vitamin D, magnesium, and zinc [9]. Intervention trials have examined the effect of single nutrient supplementation in pregnant women on HDP risk, and recently several systematic reviews and meta-analyses have synthesized the results [11-15]. To date, however, the findings do not support nutrient supplementation to reduce the risk of HDP, with the exception of calcium supplementation [16]. Calcium supplementation during pregnancy is recommended for prevention of pre-eclampsia in women with low dietary calcium intake and for those at high risk (for example, women with diabetes, renal disease, or autoimmune disease) [17,18]. Evidence from observational studies on the association between maternal nutrient intake and pre-eclampsia is inconsistent based on findings from two narrative reviews [9,10]. To our knowledge, reviews so far have summarized evidence on nutrient intake in relation to pre-eclampsia, but findings on the association between a wide range of maternal dietary factors (nutrients, foods, overall diet) and both gestational hypertension and pre-eclampsia from observational studies have not been systematically reviewed.

This study aimed to systematically review all evidence from observational studies in reproductive-age women on the associations between dietary factors including energy, nutrients, foods, and overall diet, alone or in combination with dietary supplements, and gestational hypertension and pre-eclampsia.

## Methods

### Systematic review

This review was carried out in accordance with the Meta-analysis Of Observational Studies in Epidemiology (MOOSE) guidelines [19]. A systematic search was performed using MEDLINE and EMBASE to identify relevant studies published from 1948 (MEDLINE) or 1966 (EMBASE) until the end of May 2014, using the search queries shown in Additional file 1: Table S1. The search was restricted to articles published in English and studies in human populations. Bibliographies of relevant articles and reviews were manually screened for additional potentially relevant studies.

Criteria for inclusion in this systematic review were: observational studies (including case–control and cross-sectional, retrospective, and prospective cohort studies) of women of reproductive age reporting results (in tables or text) on the association between dietary factor(s) (exposure) and gestational hypertension and/or pre-eclampsia (outcome). Dietary factors included intake of energy, nutrients, or foods, or overall dietary patterns, alone or in combination with dietary supplements. Studies were excluded if they reported on dietary supplements not in combination with dietary intake, or examined a biomarker of dietary intake.

Relevance from title and abstract was assessed based on these inclusion and exclusion criteria. If studies were considered potentially relevant, the full-text article was read. Two investigators (GDM and DAJMS) independently reviewed full-text articles based on inclusion criteria. Any disagreement was resolved by discussion.

### Data extraction

Studies identified were case–control and cohort studies, for which the following data were extracted: country, population characteristics, age, sample size, dietary exposure, dietary assessment method, validation and timing of dietary assessment, outcome, diagnostic criteria, exclusion criteria, and confounders used in analysis. For each individual dietary exposure, unadjusted and adjusted dietary intake data for cases and non-cases as well as effect estimates for the association between dietary factors and gestational hypertension and/or pre-eclampsia were extracted. If information on study characteristics was missing, definitions were unclear, or insufficient data were reported and could not be calculated (for example, missing standard deviation (SD) or confidence interval (CI), dietary intake, or number of cases), the authors were contacted for clarification or to request additional information [20-31]. A total of seven authors responded, of whom five were able provide additional information on study characteristics or results.

### Quality assessment

Two reviewers (GDM and DAJMS) independently assessed the risk of bias using the Newcastle-Ottawa Scale (NOS) for studies included in the systematic review [32]. Selection, comparability, and outcome assessment were rated for case–control and cohort studies separately. The rating-system scores studies from 0 (highest degree of bias) to 9 (lowest degree of bias).

### Meta-analysis

Extracted results were pooled in the meta-analyses when at least two studies reported uniform units of dietary intake and associated effect estimates for HDP. Results were presented using forest plots, separately for case–control and cohort studies, for each dietary exposure and each outcome (that is, gestational hypertension, pre-eclampsia, and gestational hypertension and/or pre-eclampsia). Dietary intake data were converted into uniform units when inconsistently reported across studies (kcal/day for total energy intake; mg/day for calcium, vitamin C, and sodium; g/day for n-3 fatty acids and mcg/day for vitamin D).

Using the available data, two meta-analyses were performed. 1) For case–control and cohort studies reporting unadjusted mean dietary intake with SD or standard error (SE) for cases and non-cases, the study-specific weighted mean differences (WMD) with 95% CIs were pooled, 2) For case–control and cohort studies reporting adjusted effect estimates (odds ratio (OR) or relative risk (RR)) and corresponding 95% CI for a unit increase in dietary intake or comparing the highest and lowest categories of intake), these estimates were pooled to obtain summary estimates for the associations between dietary factors and gestational hypertension and/or pre-eclampsia. ORs and RRs were combined into one meta-analysis if the incidence of the outcome was ≤10% or the effect estimate was ≥0.5 or ≤2.5 [33]. Random effects models were used, and between-study heterogeneity was assessed using the χ^2^ (Cochrane Q) and *I*^2^ statistics [34]. In cases where one large study dominated the result of a meta-analysis, this study was excluded in additional analyses to explore how this altered the pooled result. Exploring heterogeneity with subgroup analysis or meta-regression was not possible because of the limited number of studies. Publication bias was assessed via a funnel plot for meta-analyses including at least five study results. Statistical analyses were conducted using Stata software (v13.0 (Stata Corp., College Station, Texas). *P* < 0.05 was considered statistically significant.

## Results

**Figure 1:**
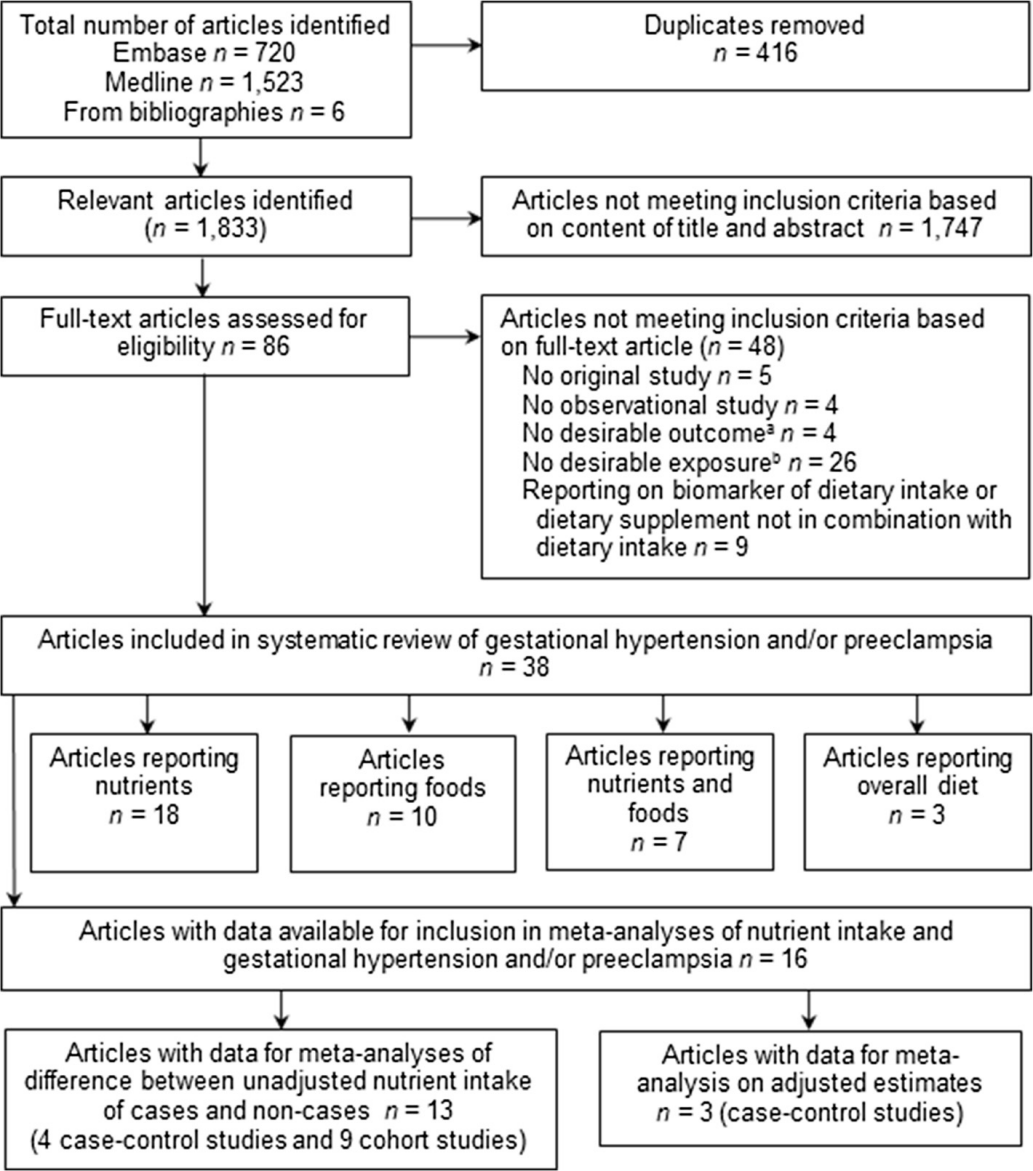
**Flow diagram for selection of studies included in systematic review and meta-analyses on the association between dietary factors and gestational hypertension and/or pre-eclampsia.** Articles reporting on both gestational hypertension and pre-eclampsia were included as two separate studies, and the number of studies suitable for pooling of results varied for the different dietary factors. **(a)** No desirable outcome, for example, combined pre-eclampsia with disorders other than gestational hypertension or examined oxidative stress or blood pressure in pregnancy; **(b)** No desirable exposure, that is, not reported on dietary intake as exposure.

The systematic literature search identified 1,833 unique articles. After screening of titles and abstracts, 86 studies were considered relevant. The full text of these articles was reviewed, and 38 met the inclusion criteria (Figure 1). Of these, 17 studies reported results that could not be pooled in meta-analyses, and were included in the systematic review only, while 21 studies reported data that could be included in meta-analyses for different dietary factors.

Four studies [20,26,35,36] reporting on the association between nutrient intake and both mild and severe pre-eclampsia were included as four separate results in meta-analyses. Another study [22] reported on dietary intake at 11–15, 26, and 34–37 weeks’ gestation. As diet assessed prior to diagnosis would best predict development of the disorder, only results on the association between diet assessed at 11–15 weeks’ gestation and development of pre-eclampsia were included in the review for this study [22]. When multiple studies on the same study population reported results for similar nutrients [20,24,37], the study with the largest number of women was included [20].

### Study characteristics

Study characteristics of the 38 included studies are presented in Table 1. Women were all recruited during pregnancy, and had a mean age between 23 and 29 years. Maternal age was comparable between cases and non-cases even in non-matched case–control studies (see Additional file 1: Table S2). In studies comparing characteristics of cases and non-cases, body mass index (BMI) and the proportion of nulliparous women were consistently higher, and gestational age at delivery and infant birth weight were consistently lower, for HDP cases compared with non-cases (see Additional file 1: Table S2). Number of pregnant women ranged from 92 to 928 in case–control studies and from 65 to 63,226 in cohort studies. The prevalence ranged from 1.7 to 17.3% for gestational hypertension, and from 1.3 to 7.6% for pre-eclampsia. Diet was assessed mostly using food frequency questionnaires (FFQs) (in 21 of 38 studies) that consisted of at least 120 food items and were validated in 16 studies. Outcome measures were collected from medical records or linkage with patient registries. In general, diagnostic criteria were consistent across studies: gestational hypertension was defined as new-onset hypertension (>140/90 mmHg) after 20 weeks’ gestation, and pre-eclampsia as gestational hypertension accompanied by proteinuria (≥300 mg/24 hours) (see Additional file 1: Table S3).

**Table 1:** **Characteristics of studies included in the systematic review**

						**Diet assessment**			
**Reference**	**Country**	**N**	**Cases, n (%)**	**Population characteristics**	**Age, years** ^**a**^	**Method**	**Period**	**Course**	**Outcome**
**Cohort studies**									
Brantsæter et al., 2011 [20]	Norway	33,399	1,755 (5.3) Mild PE: 997 (3.0) Severe PE: 514 (1.5) Unclassified PE: 244 (0.7)	Nulliparous pregnant women recruited through a nationwide postal invitation in connection with their first routine ultrasonography examination between 2002 and 2008	28.4 ± 4.4	255-item FFQ^b^	17 to 22 weeks’ gestation	Diet only	PE^c^
Goodarzi Khoigani et al., 2012 [22]	Iran	584	23 (3.9)	Pregnant women referred to 18 health centers and 12 private offices in Isfahan between 2009 and 2010	Cases: 26.48 ± 4.21; non-cases: 25.60 ± 4.44	48-hour dietary recall	11 to 15, 26, and 34 to 37 weeks’ gestation	Diet with supplements	PE
Haugen et al., 2009 [24]	Norway	23,423	1,267 (5.4)	Nulliparous pregnant women recruited through a nationwide postal invitation in connection with their first routine ultrasonography examination between 2002 and 2008	58% of women age range: 20 to 29	255-item FFQ^b^	17 to 22 weeks’ gestation	Diet with and without supplements	PE^c^
Klemmensen et al., 2009 [26]	Denmark	57,346	All subtypes: 1,487 (2.6) Severe PE: 337 (0.6)	Pregnant women recruited when first visiting their general practitioner because of pregnancy between 1996 and 2002	NR	360-item FFQ^b^	25 weeks’ gestation	Diet with supplements	PE^c^
Qiu et al., 2008 [29]	USA	1,538	64 (4.2)	Pregnant women attending prenatal care clinics affiliated with Swedish Medical Center and Tacoma General Hospital in Seattle and Tacoma, Washington between 1996 and 2002	Mean ± SE: 32.2 ± 0.1	122-item FFQ^b^	First trimester	Diet only	PE
Triche et al., 2008 [30]	USA	1,681	63 (3.7)	Pregnant women recruited between 1996 and 2000 from 56 obstetric practices and 15 clinics associated with six hospitals in Connecticut and Massachusetts	Majority of women: range: 25 to 34	Interview (chocolate foods and drinks)	Mean: 14.9 weeks’ gestation (range 6.1 to 24.3)	Diet only	PE
Saftlas et al., 2010 [31]	USA	2,508	PE: 60 (2.4) GH: 161 (6.4)	Pregnant women recruited at their first prenatal visit between 1988 and 1991 at 13 prenatal care practices in Connecticut	Majority range: 25 to 34	Interview (chocolate foods and drinks)	First and third trimester	Diet only	PE and GH
Geraldo Lopes Ramos et al., 2006 [35]	Brazil	1,052	Mild PE: 52 (4.9); severe PE: 16 (1.5); GH: 36 (3.4)	Women who gave birth at Hospital de Clínicas de Porto Alegre	Mean sge: GH cases 26.1; mild PE cases 28.1; severe PE cases 23.3; non-cases 25.2	Dietary interview	1 day after delivery (retrospective, during pregnancy)	Diet only	PE and GH
Borgen et al., 2012 [37]	Norway	32,933	1,703 (5.2)	Nulliparous pregnant women recruited through a nationwide postal invitation in connection with their first routine ultrasonography examination between 2002 and 2008	51% of women; age range: 25 to 29	255-item FFQ^b^	17 to 22 weeks’ gestation	Diet only	PE^c^
Brantsæter et al., 2009 [38]	Norway	23,423	1267 (5.4)	Nulliparous pregnant women recruited through a nationwide postal invitation in connection with their first routine ultrasonography examination between 2002 and 2008	58% of women; age range: 20 to 29	255-item FFQ^b^	17 to 22 weeks’ gestation	Diet only	PE^c^
Chavarro et al., 2011 [39]	Denmark	63,226	2,206 (3.49)	Pregnant women recruited when first visiting their general practitioner because of pregnancy between 1998 and 2003	29 ± 4	360-item FFQ^b^	25 weeks’ gestation	Diet only	PE
Clausen et al., 2001 [40]	Norway	3,133	85 (2.7); early-onset PE: 27 (0.9); late-onset PE: 58 (1.8)	Caucasian pregnant women, representing all socioeconomic classes, delivering between 1994 and 1996 at Aker Hospital in Oslo	Cases 29.2 ± 4.9; non-cases 29.9 ± 4.5	180-item FFQ^b^	Early second trimester	Diet with supplements	PE
Longo-Mbenza et al., 2008 [41]	Congo	238	PE: 7 (2.9) GH: 4 (1.7)	Pregnant women admitted to the Evangelical Hospital of Kimpese, located in a rural area, between 2002 and 2003	Cases: 25.5 ± 7.2; non-cases: 27.4 ± 6.4	Questionnaire (vegetables and meat)	First trimester	Diet only	PE and/or GH
Morris et al. 2001 [42]	USA	4,314	PE : 326 (7.6) GH: 747 (17.3)	Nulliparous pregnant women enrolled in a randomized clinical trial seeking prenatal care at university medical centers and affiliated clinics/hospitals in five communities	84% of women mean: <25	24-hour dietary recall	13 to 21 weeks’ gestation	Diet with supplements	PE^c^ and GH
Oken et al., 2007 [43]	USA	1,718	PE: 59 (3) GH: 119 (7)	Pregnant women recruited at eight offices of Harvard Vanguard Medical Associates, a large multispecialty urban/suburban group practice in eastern Massachusetts, at first prenatal visit between 1999 and 2002	~90% of women age range: 20 to 40	>140-item FFQ^b^	First trimester	Diet with and without supplements	PE and GH
Olafsdottir et al., 2006 [44]	Iceland	488	PE: 19 (3.9) GH: 30 (6.1)	Randomly selected pregnant women, attending a routine first visit at the Center of Prenatal Care in Reykjavik from 1999 to 2001	GH cases 29 ± 6; PE cases; 26 ± 4; non-cases: 28 ± 5	150-item FFQ^b^	11 to 15 weeks’ gestation	Diet with supplements	PE and/or GH
Ortega et al., 1999 [45]	Spain	82	6 (7.3)	Pregnant women who were to deliver at the Cuenca INSALUD Hospital in Cuenca city area between 1990-1991	Non-cases: 27.0 ± 3.9; cases; 26.2 ± 3.4	5-day dietary record	Third trimester	Diet with and without supplements	GH
Richardson et al., 1995 [46]	USA	9,291	83 (3.8) (black women) and 185 (2.6) (white women)	Pregnant women who are members of a prepaid medical insurance plan, residing in the Oakland area of California and who delivered between 1959 and 1967	Majority range: 20 to 34	Interview (glasses of milk per day)	During pregnancy	Diet with and without supplements	PE
Rifas-Shiman et al., 2009 [47]	USA	1,777	60 (3.4)	Pregnant women recruited at eight offices of Harvard Vanguard Medical Associates, a large multispecialty urban/suburban group practice in eastern Massachusetts, at first prenatal visit between 1999 and 2002	64% of women range: 25 to 35	166-item FFQ^b^	First and second trimester	Diet only	PE
Rumbold et al., 2005 [48]	Australia	229	38 (12) GH or PE, 20 (7) GH, 17 (5) PE	Women attending the antenatal clinic of the Women’s and Children’s Hospital for routine antenatal care in Adelaide between April and July 2001	Cases: 28 ± 5 cases; non-cases: 28 ± 5	166-item FFQ^b^	Mid to late pregnancy	Diet with supplements	PE or GH
Skajaa et al., 1991 [49]	Denmark	965	13 (1.3)	Women recruited when attending one of two antenatal clinics in Aarhus between 1988 and 1989	Median (range): 28.7 (18 to 45)	Interview	30 weeks’ gestation (retrospective, previous 3 months)	Diet with supplements	PE
Tande et al., 2013 [50]	USA	65	13 (20)	Nulliparous pregnant women recruited at the time of their first prenatal visit at a local obstetrics clinic	Mean ± SE: non-cases: 24.2 ± 0.62; cases: 25.3 ± 0.72	78-item FFQ	<14 weeks’ gestation	Diet with supplements	PE or GH
Timmermans et al., 2011 [51]	Netherlands	3,187	PE: 58 (1.8) GH: 165 (5.2)	Pregnant women living in Rotterdam delivering between 2002 and 2006	31.6 ± 4.0	293-item FFQ^b^	Early pregnancy (median 13.5 wks, IQR 3.4)	Diet only	PE and GH
**Case–control studies**									
Frederick et al., 2005 [21]	USA	511	172 cases, 339 controls	Women delivering at Swedish Medical Center and Tacoma General Hospital in Washington between 1998 and 2001. Controls were normotensive women, delivering on the same day as a case, matched for parity and maternal age	Mean ± SEM: cases: 29.9 ± 0.5; controls: 30.6 ± 0.3	121-item FFQ^b^	During postpartum hospital stay (retrospectively, 12 months prior to delivery)	Diet only	PE
Gulsen et al., 2012 [23]	Turkey	247	92 cases, 155 controls	Pregnant women from Konya and neighboring cities hospitalized with pre-eclampsia at a clinic between 2004 and 2005. Controls were healthy pregnant women visiting the same institute for routine control	Range: 20 to 34: 65% of cases; 88% of controls	Questionnaire (7 food groups)	During pregnancy	Diet only	PE
Kesmodel et al., 1997 [25]	Denmark	764	PE 43 cases, 256 controls GH: 179 cases, 256 controls	Population-based nested case–control study of women who delivered at Aarhus University hospital between 1989 and 1991. Controls were evenly distributed over and covering all months of the period corresponding to the recall time in the case groups	NR	FFQ	Postpartum (retrospective, during pregnancy)	Diet with supplements	PE and GH
Marcoux et al., 1991 [27]	Canada	928	PE: 172 (505 controls) GH: 251 (505 controls)	Pregnant women who delivered in Quebec City or Montreal between 1984 and 1986. Controls were women who delivered immediately after the case in the same hospital and had not more than one elevated blood pressure reading after 20 weeks of pregnancy	PE cases: 26.0 ± 4.8; GH cases: 26.2 ± 4.3; controls: 26.1 ± 4.2	20-item FFQ (dairy foods only)	After delivery (retrospective, first 20 weeks’ gestation)	Diet only	PE^c^ and GH
Paknahad et al., 2008 [28]	Iran	92	46 cases, 46 controls	Pregnant women attending Al-Zahra and Shaheed Beheshti hospitals in Isfahan. Controls were normotensive pregnant women matched for age and parity	Mean ± SE: cases: 26.5 ± 0.89; controls: 24.6 ± 0.72	FFQ and 24-hour dietary recall	Mean ± SD 33.7 ± 2.7 weeks’ gestation	Diet only	PE or GH
Wei et al., 2009 [36]	Canada	337	92 cases (69 severe PE; 23 mild PE), 245 controls	Nulliparous pregnant women recruited within 48 hours after delivery in four hospitals in Quebec. Controls were normotensive pregnant women delivering during the same period as the case	Cases: 29.0 ± 5.2; controls: 29.1 ± 5.3	Questionnaire	Within 48 hours after delivery	Diet only	PE
Al et al., 1994 [52]	Netherlands	116	29 cases, 87 controls	Nested case–control study of pregnant women recruited at hospitals in the Maastricht region. Cases were matched for parity and hospital with controls with an uncomplicated pregnancy who delivered around the same time	Mean ± SEM: controls: 28.5 ± 0.35; cases: 27.9 ± 0.54	FFQ and diet history	22 weeks’ gestation	Diet only	PE
Atkinson et al., 1998 [53]	Zimbabwe	374	180 cases, 194 controls	Pregnant women delivering at one of nine clinics from the Harare Maternity Hospital located in suburbs of Harare city, between 1995 and 1996. Most patients were from poor urban areas or migrated between rural and urban areas. The first healthy women admitted after each case was used as a control	Cases: 25.6 ± 6.4; controls: 24.8 ± 7.9	Questionnaire (meat, poultry, fruit, fish, vegetables, and dairy)	During postpartum hospital stay (retrospectively, month prior to birth)	Diet only	PE^c^
Duvekot et al., 2002 [54]	Netherlands	NR	163 cases	Pregnant women selected from a computer database and patient charts in two hospitals between 1991 and 1996. Controls were matched for age and delivery date	Median ± SD: cases: 28 ± 1; controls: 28 ± 0.3	Questionnaire (milk consumption and calcium supplement use)	During pregnancy	Diet with supplements	PE^c^
Kazemian et al., 2013 [55]	Iran	263	113 cases, 150 controls	Pregnant women referred to Shahid Akbarabadi Hospital between January and May 2011. Controls were pregnant women with normal blood pressure referred to this hospital for prenatal care, matched for gestational age	Cases: 28.73 ± 6.04; controls: 25.36 ± 4.84	148-item FFQ^b^	At diagnosis (retrospectively, past 3 months)	Diet with supplements	GH
Reyes et al., 2012 [56]	Colombia	402	201 cases, 201 controls	Pregnant women recruited from six Colombian cities between 2006 and 2009. Healthy pregnant controls were matched for age and selected from the same city of residence and the same hospital of delivery as the case	Cases: 26.45 ± 7.22; controls: 26.71 ± 7.21	90-item FFQ^b^	Before delivery (retrospective, last 12 months)	Diet only	PE
Richards et al., 2014 [57]	South Africa	192	96 cases, 96 controls	Women who delivered at the Maternity Centre at Groote Schuur Hospital and Mowbray Maternity Hospital in Cape Town between January and November 2010. Healthy pregnant women who delivered a live infant were matched with cases by ethnicity, gravidity, age, and gestational age at delivery	Cases: 24 ± 4.3; controls: 24 ± 4.4	Questionnaire	After delivery	Diet only	PE
Schiff et al., 1996 [58]	USA	138	48 cases, 90 controls	Pregnant women admitted to the EH Crump Women’s Hospital in Memphis, Tennessee between January 1994 and April 1995. Normal outpatients with no evidence of hypertension or proteinuria either at recruitment or delivery in the third trimester served as controls	Cases: 21.5 ± 5.4; controls: 20.1 ± 4.4	>100-item questionnaire	During pregnancy	Diet with and without supplements	PE^c^
Sharbaf et al., 2013 [59]	Iran	140	40 cases, 100 controls	Nulliparous pregnant women recruited within 48 hours after delivery in two hospitals in Tehran. Controls were normotensive pregnant women delivering during the same time as the case	Cases: 28 ± 4.1; controls: 27 ± 5	Questionnaire	Within 48 hours after delivery	Diet only	PE
Zhang et al., 2002 [60]	USA	368	109 cases, 259 controls	Women delivering at Swedish Medical Center and Tacoma General Hospital in Washington between 1998 and 2000. Controls were normotensive women, delivering on the same day of a case	Mean ± SEM: : cases: 31.1 ± 0.6; controls: 29.9 ± 4.5	121-item FFQ^b^	During postpartum hospital stay (retrospectively, 12 months prior to delivery)	Diet only	PE

FFQ, food frequency questionnaire; GH, gestational hypertension; NR, not reported; PE, pre-eclampsia; SD, standard deviation; SE, standard error; SEM, standard error of the mean.

^a^Age reported as mean ± SD unless indicated.

^b^Validated dietary assessment.

^c^Pre-eclampsia/eclampsia.

### Quality assessment

Quality assessment ratings and scores are shown in Additional file 1: Table S4. Total scores ranged from 3 to 8 for case–control studies and from 4 to 9 for cohort studies, out of a maximum score of 9. Case definition and outcome assessment were of high quality and defined using hospital records in the majority of studies. In addition, most cohort studies used dietary recall or a validated FFQ to assess dietary intake. Main concerns included 1) comparability of cases and non-cases on basis of design or analysis (the majority of case–control studies and half of the cohort studies did not match or adjust for important confounding factors including maternal age, hypertension prior to pregnancy, and parity); 2) exposure ascertainment in case–control studies (most studies used non-validated general questionnaires to assess dietary intake); and 3) representativeness of the study samples (no information on non-response rates in case–control studies, and selection bias or insufficient information on derivation of the sample in most cohort studies).

### Associations between nutrient intake and hypertensive disorders of pregnancy

Results on differences in nutrient intake between HDP cases and non-cases are shown in Additional file 2, and associations between nutrient intake and HDP adjusted for confounding factors in Additional file 3. Results for most nutrients were sparse, inconsistent, or not statistically or clinically significant (Additional files 2 and 3, meta-analyses results not shown), with the exception of total energy, magnesium, and calcium intake which are described in more detail below.

#### Total energy intake

**Figure 2:**
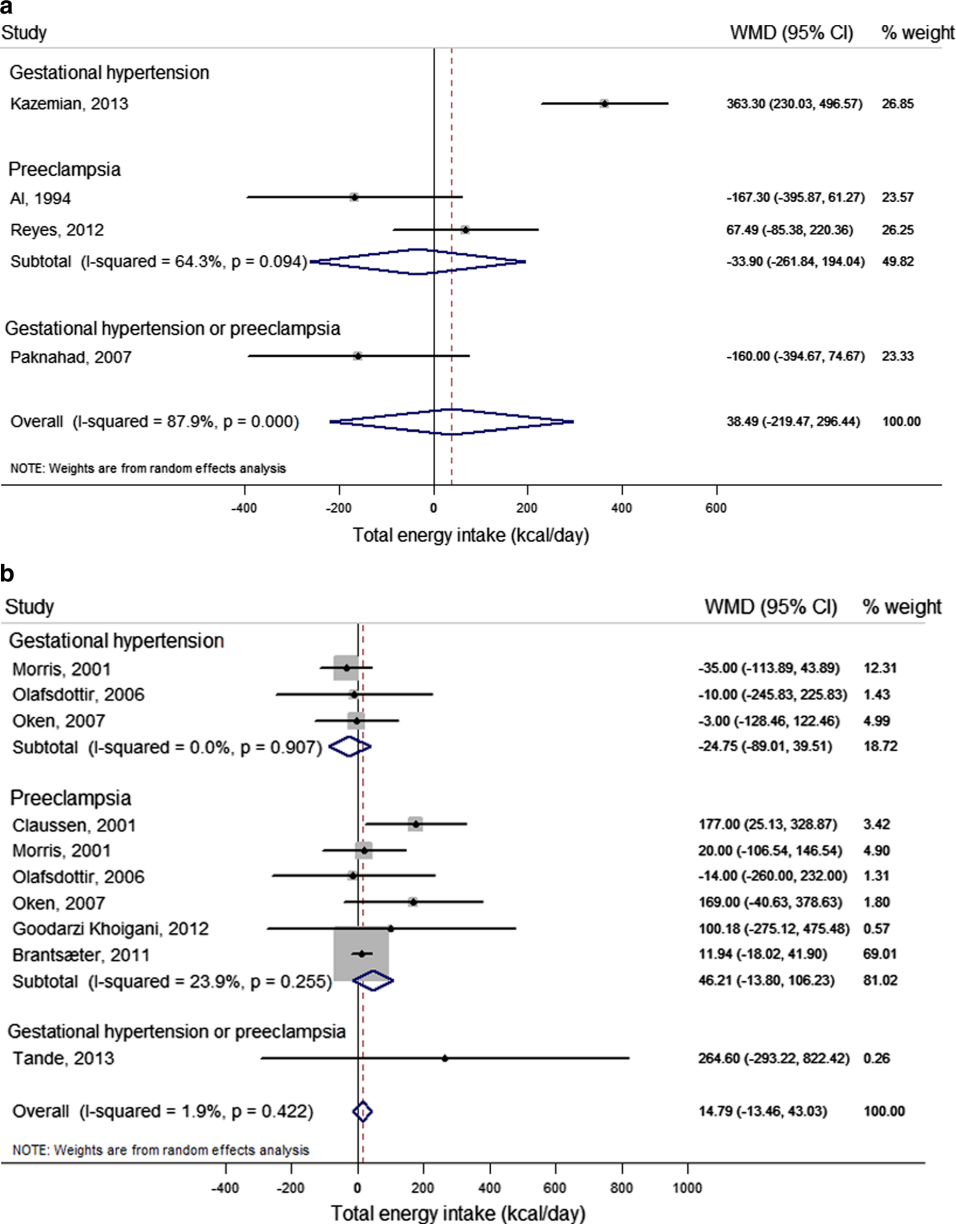
**Difference in unadjusted total energy intake (kcal/day) between cases (gestational hypertension, pre-eclampsia and gestational hypertension or pre-eclampsia) and non-cases (reference) reported in case–control studies and cohort studies.** For each study, the center of each square indicates the weighted mean difference (WMD), and the horizontal line indicates the 95% confidence interval; the area of the square is proportional to the weight that the individual study contributes to the overall pooled mean difference; and the diamonds are pooled mean differences (for each outcome and overall). **(a)** Case–control studies. Meta-analysis of 4 studies from 4 articles with data from 873 pregnant women, including 113 gestational hypertension, 230 pre-eclampsia, and 46 gestational hypertension or pre-eclampsia cases. **(b)** Cohort studies. Meta-analysis of 10 studies from 7 articles with data from 43,701 pregnant women, including 896 gestational hypertension, 2,267 pre-eclampsia, and 13 gestational hypertension or pre-eclampsia cases.

Five case–control studies [21,28,52,55,56] and ten cohort studies [20,22,24,37,40,42-44,49,50] reported on the difference in unadjusted total energy intake between women with and without HDP (Additional file 2). Of these, four case–control studies [28,52,55,56] and seven cohort studies [20,22,40,42-44,50] were included in the meta-analysis (Figure 2a and b, respectively). Results from case–control studies were inconsistent, and did not show an association between energy intake and HDP. Findings from cohort studies indicated that pre-eclampsia cases reported an energy intake of 46 kcal/day higher than women without pre-eclampsia (95% CI −13.80 to 106.23; *I*^2^ = 23.9%, *P =* 0.26), although this was not statistically significant. As the result for pre-eclampsia was dominated (weight 69%) by findings from a large prospective cohort, the Norwegian Mother and Child Cohort Study (MoBa) [20], we additionally present the forest plot excluding this study (see Additional file 1: Figure S1). With this exclusion, the difference in total energy intake between pre-eclampsia cases and non-cases became slightly larger and statistically significant (87 kcal/day, 95% CI 5.99 to 168.11; *I*^2^ = 0.0%, *P* = 0.45). Exclusion of the MoBa study did not alter the overall non-significant result for HDP.

Only two studies reported multivariate results [40,55] (Additional file 3; see Additional file 1: Table S5), that could not be pooled because of different units of exposure. Results from a Norwegian prospective cohort study [40] showed higher odds for developing pre-eclampsia with higher early second trimester energy intake (OR = 3.7, 95% CI 1.5 to 8.9, highest versus lowest quartile). Kazemian *et al.* [55] reported a positive association between higher energy intake and gestational hypertension in a case–control study (OR = 1.33, 95% CI 1.17 to 1.52, per 200 kcal).

#### Magnesium

**Figure 3:**
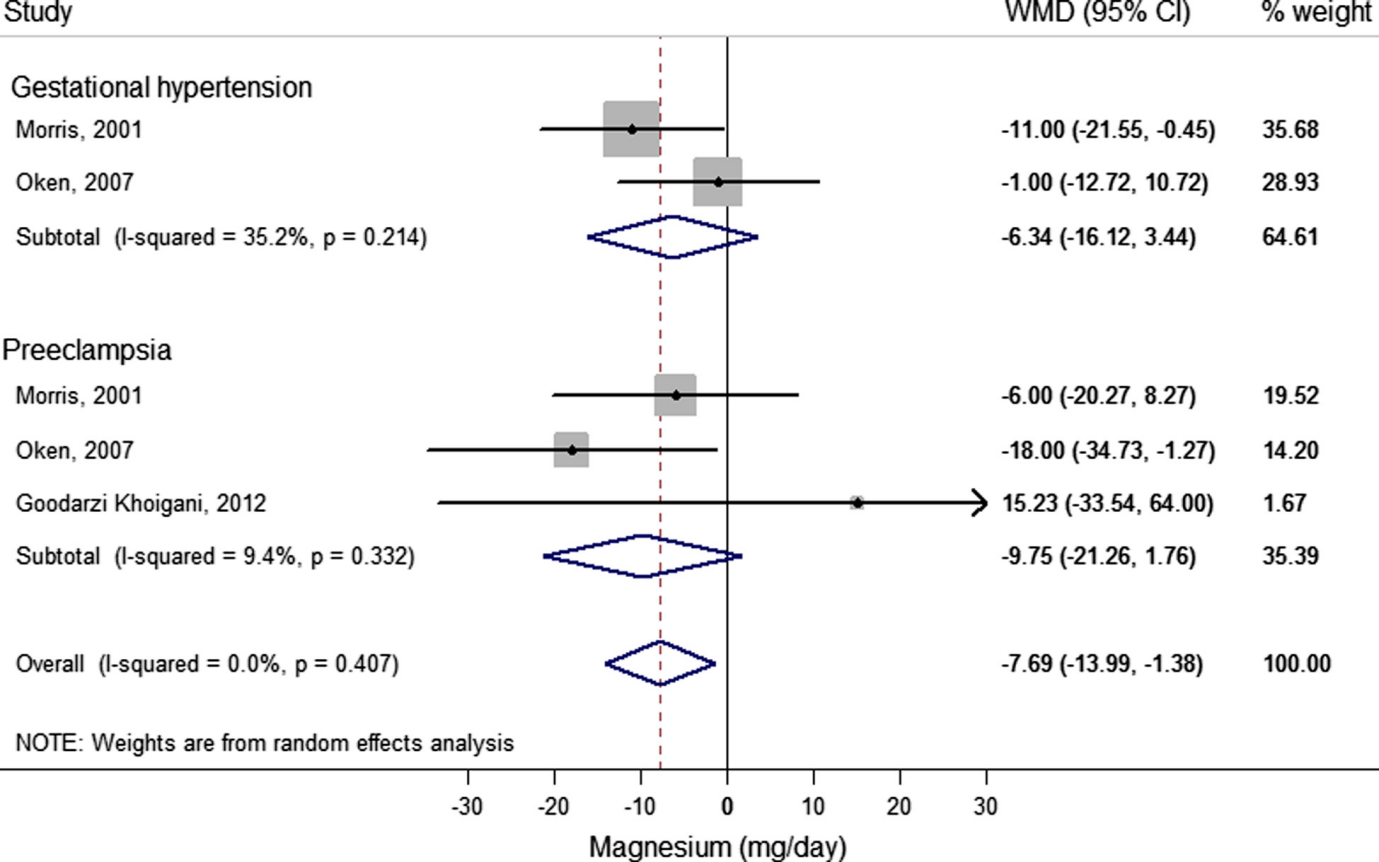
**Difference in unadjusted magnesium intake (mg/day) between cases (gestational hypertension and pre-eclampsia) and non-cases (reference) reported in cohort studies.** For each study, the center of each square indicates the weighted mean difference (WMD), and the horizontal line indicates the 95% confidence interval; the area of the square is proportional to the weight that the individual study contributes to the overall pooled mean difference; and the diamonds are pooled mean differences (for each outcome and overall). Meta-analysis of 5 studies from 3 articles with data from 6,616 pregnant women, including 866 gestational hypertension and 408 pre-eclampsia cases.

Two case–control studies [21,55] and four cohort studies [22,42,43,49] reported on unadjusted magnesium intake in women with and without HDP (Additional file 2), of which three cohort studies [22,42,43] could be included in the meta-analysis (Figure 3). Pooled results revealed statistically significantly lower mean magnesium intake of 8 mg/day for women with HDP (95% CI −13.99 to −1.38; *I*^2^ = 0.0%, *P* = 0.41).

Five studies [21,42,43,49,55] reported multivariate results for the association between magnesium intake and HDP (Additional file 3; see Additional file 1: Table S5). Estimates could not be pooled in meta-analysis because of different units of exposure. Studies consistently trended towards an inverse association between magnesium intake and gestational hypertension [42,43,55] and pre-eclampsia [21,42,43,49], although this was not statistically significant (Additional file 3).

#### Calcium

**Figure 4:**
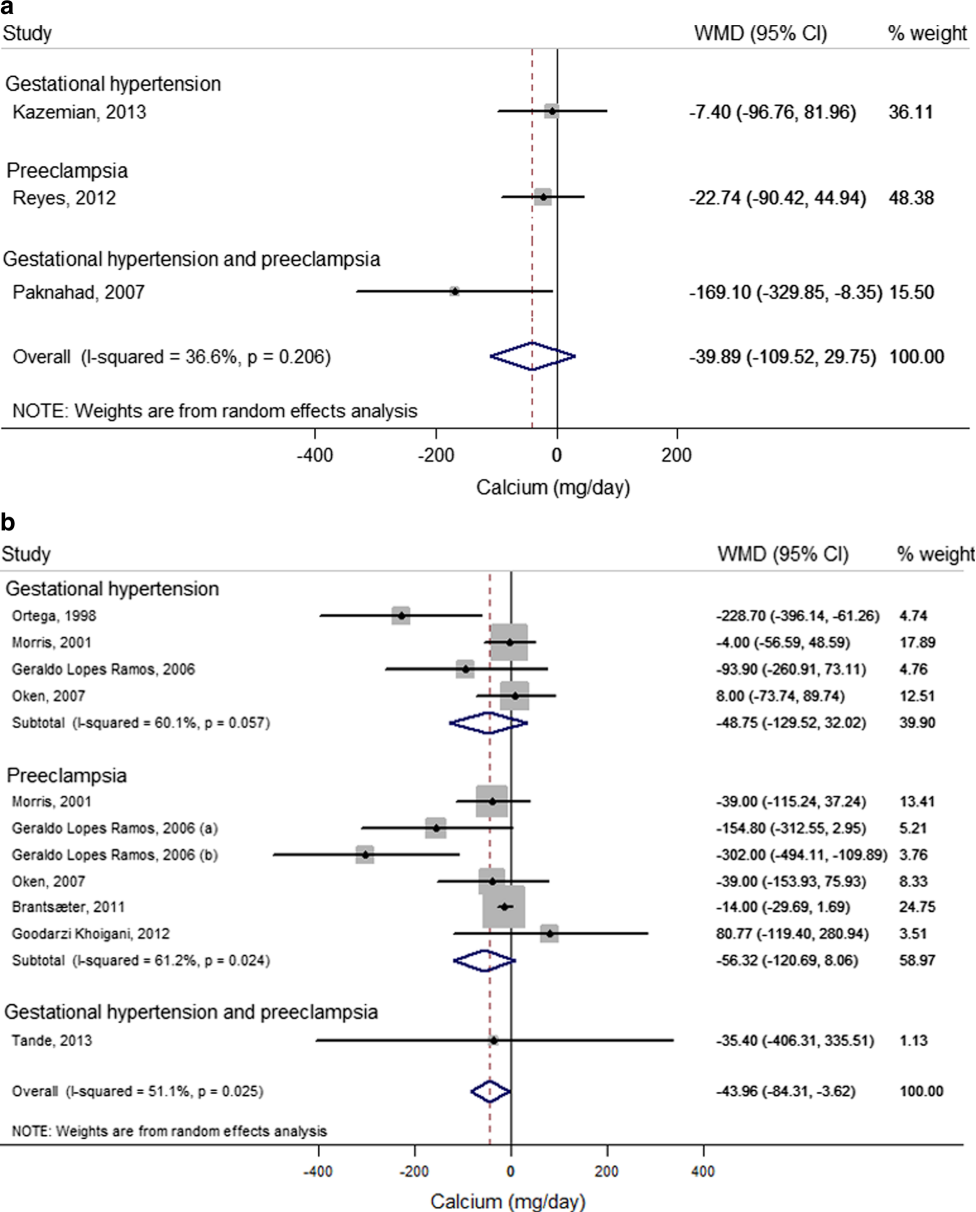
**Difference in unadjusted calcium intake (mg/day) between cases (gestational hypertension, pre-eclampsia and gestational hypertension or pre-eclampsia) and non-cases (reference) reported in case–control and cohort studies.** For each study, the center of each square indicates the weighted mean difference (WMD) and the horizontal line indicates the 95% confidence interval; the area of the square is proportional to the weight that the individual study contributes to the overall pooled mean difference; the diamonds are pooled mean differences (for each outcome and overall). **(a)** Case–control studies. Meta-analysis of 3 studies from 3 articles with data from 757 pregnant women, including 113 gestational hypertension, 201 pre-eclampsia, and 46 gestational hypertension or pre-eclampsia cases. **(b)** Cohort studies. Meta-analysis of 11 studies from 7 articles with data from 41,214 pregnant women and 908 gestational hypertension, 2,231 pre-eclampsia and 13 gestational hypertension or pre-eclampsia cases. **(a)** Mild pre-eclampsia; **(b)** severe pre-eclampsia (for definition, see Additional file 1: Table S3).

Of seven case–control studies [21,25,27,28,35,55,56] and six cohort studies [20,22,42,43,45,50] reporting on unadjusted calcium intake in HDP cases and non-cases, three case–control studies [28,55,56] and seven cohort studies [20,22,35,42,43,45,50] could be included in the meta-analysis (Figure 4a and b, respectively). Results from case–control studies consistently showed lower reported calcium intake for HDP cases compared with non-cases, but the pooled result was not statistically significant (WMD = −39.89, 95% CI −109.52 to 29.75; *I*^2^ 36.6%; *P* = 0.21). Results from cohort studies showed borderline significantly lower reported mean calcium intake of 56 mg/day (95% CI −120.69 to 8.06) for pre-eclampsia cases compared with non-cases, with moderate between-study heterogeneity (*I*^2^ = 61.2%, *P* = 0.02). An overall mean difference of 44 mg/day was found for women with HDP (95% CI −84.31 to −3.62) with significant between-study heterogeneity (*I*^2^ = 51.1%, *P* = 0.03).

**Figure 5:**
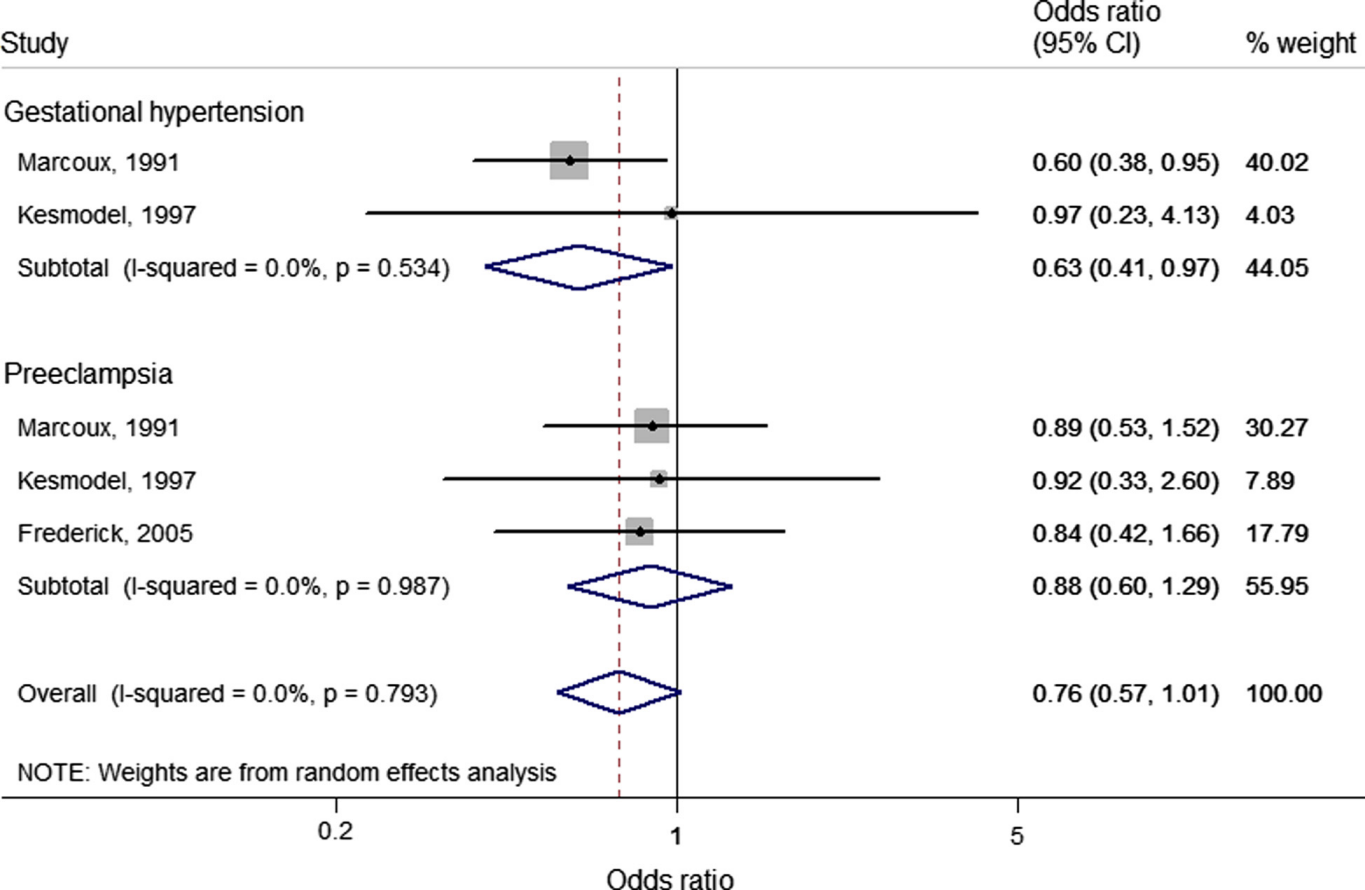
**Adjusted association between calcium intake (highest versus lowest category of intake (reference) and hypertensive disorders of pregnancy based on case–control studies.** Meta-analysis of 5 studies from 3 articles with data from 2,203 pregnant women, including 430 gestational hypertension and 387 pre-eclampsia cases. For each study, the center of each square indicates the odds ratio, and the horizontal line indicates the 95% confidence interval; the area of the square is proportional to the weight that the individual study contributes to the overall pooled odds ratios; and the diamonds are pooled odds ratios (for each outcome and overall).

Four case–control studies [21,25,27,55] and two cohort studies [42,43] reported adjusted estimates for the association between calcium intake and HDP (Additional file 3; see Additional file 1: Table S5), of which three case–control studies [21,25,27] could be included in the meta-analysis (Figure 5). Calcium intake in the highest (>1600 mg/day approximately) compared with the lowest (<1000 mg/day approximately) quintile consistently showed lower odds for gestational hypertension (OR = 0.63, 95% CI 0.41 to 0.97; *I*^2^ = 0.0%, *P* = 0.53) and overall HDP (OR = 0.76, 95% CI 0.57 to 1.01; *I*^2^ = 0.0%, *P* = 0.79).

Interpretation of the funnel plots for the association between energy, magnesium and calcium and HDP showed no suggestion of publication bias (*P*-values for Egger's test for small-study effects all >0.05, data not shown).

### Associations between food groups/dietary patterns and hypertensive disorders of pregnancy

Results for unadjusted and adjusted associations between food groups and overall dietary patterns and HDP suggested beneficial effects of fruit and vegetable consumption (Additional file 4). These studies could not be pooled in a meta-analysis because of differences in the foods or patterns examined or different units of exposure.

#### Fruit and vegetables

Six case–control [21,23,53,56,57,60] and four cohort studies [26,37,40,41] examined the association between fruit and/or vegetable consumption and pre-eclampsia (Additional file 4). Two case–control studies (based on one study population) [21,60] and two cohort studies [26,37], all adjusting for confounding factors (see Additional file 1: Table S5), consistently suggested a beneficial effect of higher fruit and/or vegetable consumption on pre-eclampsia, although they were not all statistically significant.

#### Dietary patterns

Only three studies reported on the associations between overall dietary patterns and HDP [38,47,51]. In the MoBa study (23,423 women, including 1,267 pre-eclampsia cases) [38] inverse associations were found with development of pre-eclampsia in women with high scores on a dietary pattern (identified by factor analysis) characterized by vegetables, plant foods, and vegetable oils (third versus first tertile OR = 0.72, 95% CI 0.62 to 0.85), and higher odds of pre-eclampsia were found in women with a dietary pattern characterized by processed meat, salty snacks, and sweet drinks (OR = 1.21, 95% CI 1.03 to 1.42). Studies by Timmermans *et al*. [51] (3,187 women, including 165 gestational hypertension and 58 pre-eclampsia cases) and Rifas-Shiman *et al*. [47] (1,777 women, including 60 pre-eclampsia cases) were smaller and less conclusive. In the Generation R study [51], an association was found between low adherence to a Mediterranean-style dietary pattern and high adherence to a traditional dietary pattern, identified by factor analysis and higher blood pressure during pregnancy, but these patterns were not associated with gestational hypertension or pre-eclampsia outcomes. In the US cohort study Project Viva [47], diet quality, as measured by the Alternate Healthy Eating Index slightly modified for pregnancy (AHEI-P), was not associated with pre-eclampsia (OR = 0.96, 95% CI 0.84 to 1.10, 5 point increase) when assessed in the first trimester, but slightly lowered the odds of developing pre-eclampsia when assessed in the second trimester of pregnancy (OR = 0.87, 95% CI 0.76 to 1.00).

## Discussion

This study shows the sparse body of evidence from observational studies published on the associations between dietary factors and HDP. Meta-analyses of cohort studies showed higher unadjusted reported energy intake (46 kcal/day) for pr-eeclampsia cases and lower intake of magnesium (8 mg/day) and calcium (44 mg/day) for HDP cases, compared with non-cases. Meta-analysis of multivariable results showed an inverse association between calcium intake and both gestational hypertension and overall HDP. Systematic review of a few studies examining foods and dietary patterns suggests a beneficial effect on pre-eclampsia of a diet rich in fruit and vegetables.

To our knowledge, this is the first systematic review and meta-analysis of observational studies examining the association between dietary factors and gestational hypertension or pre-eclampsia. Our review covered a wide range of dietary factors, including intake of total energy, nutrients, foods, and overall dietary patterns. Intervention studies have examined supplementation of single nutrients during pregnancy; however, evidence from observational studies of a range of dietary factors representing habitual intake may contribute to development of practical dietary guidelines for pregnant women.

Our study also has several limitations. There was substantial heterogeneity beween studies examining differences in calcium intake between women with and without HDP, which could not be further explored by subgroup analysis because of the limited number of studies. Possible explanations include differences in population characteristics (ethnicity, nutrient deficiencies, economic development, lifestyle), HDP severity, or dietary assessment methods and timing (prior to or after diagnosis). Moreover, causal relationships cannot be inferred from observational studies. Randomized controlled intervention trials are more likely to minimize confounding; however, it is practically impossible to conduct long-term controlled trials examining intake of a range of nutrients and foods or overall diets as the exposure.

The quality of the present review is determined by the validity of the individual studies included. Firstly, diet was assessed using a validated FFQ in 16 of 38 studies; however, questionnaires were not validated for use in pregnant women in most studies. The presence of random and systematic measurement errors in self-reported dietary intake could attenuate the associations found, and reduce the statistical power to detect an association. In addition, timing of dietary assessment was not prior to, but at or after diagnosis in case–control studies [21,23,25,27,28,36,52-60] and two retrospective cohort studies [35,49], which may have caused recall bias. Additionally, dietary assessment methods differed between studies (FFQs, dietary interviews, recalls, or records). A dietary recall may more accurately assess actual nutrient intake compared with an FFQ; however, possible heterogeneity in pooled results due to different dietary assessment methods used could not be formally tested because of the limited number of studies. Secondly, even though results from unadjusted meta-analysis were generally consistent with study results adjusted for confounding factors, studies may have failed to control for key confounding factors. Most studies adjusted for maternal age, parity, BMI, smoking, socioeconomic status, and total energy intake, and some adjusted for ethnicity and other dietary factors, but very few adjusted for factors such as HDP in previous pregnancy, or multiple pregnancy, gestational age, and physical activity, which are important determinants of HDP [3]. Furthermore, HDP are heterogeneous, and there may be etiological differences according to severity and timing of onset between disorders; however, few studies reported results for these subtypes separately. Four studies examining subtypes suggested a more pronounced risk for severe compared with mild pre-eclampsia for lower intake of calcium [35], vitamin C [26], and probiotics [20], and higher consumption of tea [36]. Clausen *et al.* found a significant trend towards increasing intake of energy and sucrose across categories without pre-eclampsia, late-onset pre-eclampsia, and early-onset pre-eclampsia [40]. The magnitude of associations between dietary factors and HDP might therefore not be generalizable to all subtypes of HDP. In meta-analysis of cohort studies on difference in calcium intake between HDP cases and non-cases, only the study by Geraldo Lopes Ramos *et al.* reported on mild and severe pre-eclampsia separately, showing a stronger and statistically significant association of lower calcium intake with severe pre-eclampsia compared with mild pre-eclampsia [35]. Exclusion of the result on severe pre-eclampsia from meta-analysis reduced the significant between-study heterogeneity for pre-eclampsia and HDP, as well as the statistically significant overall results of lower calcium intake for HDP cases compared with non-cases. Even though not all results were statistically significant, the mostly consistent direction of lower magnesium and calcium intake for HDP cases compared with non-cases may indicate overlapping pathophysiological mechanisms for dietary factors influencing gestational hypertension and pre-eclampsia, but this requires further research.

Several mechanisms could explain the associations found between dietary factors and HDP. The higher energy intake for women with HDP compared with women without HDP may reflect an imbalance between energy intake and expenditure, which could lead to overweight/obesity, a potential risk factor for HDP.

The lower reported magnesium intake for HDP cases compared with non-cases is in line with lower serum magnesium levels found in women with pre-eclampsia in some studies [10]. Magnesium may lower blood pressure by changing nitric oxide synthesis [61]. In addition, it has been suggested that lower magnesium intake may reduce the prostacyclin:thromboxane ratio, and thereby influence HDP [62].

We also observed lower reported calcium intake for women with HDP compared with women without HDP. Although this was not statistically significant after adjustment for confounding factors, studies consistently showed decreased odds for pre-eclampsia with higher calcium intake. It has been hypothesized that calcium influences HDP by reducing parathyroid hormone concentration, leading to lower intracellular free calcium levels, which results in smooth muscle contractility and vasoconstriction [63]. Calcium has also been shown to affect uteroplacental and fetoplacental blood flow by reducing resistance in the uterine and umbilical arteries [64].

Studies in this review examining foods and dietary patterns suggested a beneficial effect on pre-eclampsia of a diet high in fruit and/or vegetables. Inflammation and endothelial dysfunction may play a role in the development of HDP, and lower concentrations of inflammatory markers have been found to be associated with consumption of a diet rich in fruit and vegetables [65,66]. Fruit and vegetables are low in fat and calories, and are important sources of nutrients related to hypertension in non-pregnant populations including dietary fiber, calcium, magnesium, potassium, and vitamins C [67].

Apart from intake of total energy, magnesium, calcium, and fruit and vegetables, consumption of other nutrients and foods were not associated with HDP. This may be due to the studies including low-risk populations in which nutrient deficiencies are rare and women regularly take multivitamin supplements [68], or due to lack of heterogeneity of intake in most well-nourished populations, reducing the ability to detect an association with HDP [42]. Further studies in different populations are needed to examine a range of nutrients and foods in relation to HDP.

Reported differences in total energy, calcium, and magnesium intake between gestational hypertension or pre-eclampsia cases and non-cases were small, suggesting that small changes in intake of these nutrients would suppress any differences. Reducing total energy intake and increasing intakes of magnesium and calcium are in line with national dietary guidelines [69] promoting a healthy weight and consuming more fruits, vegetables, and fat-free and low-fat dairy products.

In line with our findings, two narrative reviews both concluded that evidence on the role of diet and HDP is very limited, with no compelling evidence from intervention or observational studies for an association between maternal nutrient intake or nutrient supplementation and pre-eclampsia risk, with the exception of calcium supplementation in high-risk populations and in women with calcium deficiency [9,10]. In the majority of studies included in meta-analysis of WMD of calcium intake, the reported intake was in line with the recommended intake (>1000 mg/day [70]), with the exception of three studies [22,35,45]. These studies showed the largest difference in calcium intake between HDP cases and non-cases, even though not all were statistically significant. Only the studies by Ortega *et al.* [45] and Geraldo Lopes Ramos *et al.* [35] showed statistically significantly lower calcium intake for cases compared with non-cases. Adherence to the recommended calcium intake may therefore have contributed to between-study heterogeneity even though this could not be formally tested. This finding is in line with results from randomized controlled trials showing reduced pre-eclampsia risk with calcium supplementation only in populations with low calcium intake. Meta-analysis of adjusted estimates, however, showed borderline significant findings of a reduced HDP risk with higher calcium intake (OR = 0.76, 95% CI 0.57 to 1.01) in study populations with reported calcium intake >1000 mg/day [21,25,27]. In addition to calcium, results from this systematic review and meta-analysis suggest a role for total energy and magnesium intake in the development of HDP, as well as a beneficial effect on pre-eclampsia of a diet rich in fruit and vegetables. Consistent with results from randomized controlled trials on nutrient supplementation, our results did not show associations between pre-eclampsia and reported intakes of vitamin D, C, and E, and n-3 polyunsaturated fatty acids [11-13]. A recent meta-analysis of dietary intervention studies showed a significant effect of dietary counseling on maternal blood pressure (systolic blood pressure: standardized mean difference −0.26, 95% CI −0.45 to −0.07; *I*^2^ = 0%, *P* = <0.001, three studies; and diastolic blood pressure: standardized mean difference −0.57, 95% CI −0.75 to −0.38; *I*^2^ = 0%, *P* = <0.001, three studies), but not HDP outcomes [71].

## Conclusions

Results from this systematic review and meta-analysis indicate that current evidence from observational studies on the association between dietary factors and HDP is limited. The short-term and long-term adverse health outcomes for both mother and offspring associated with HDP highlight the importance of identification of preventive strategies. Based on the cohort studies included in this review, maternal dietary intake of total energy was higher for pre-eclampsia cases compared with non-cases, although this was not statistically significant. In line with existing guidelines, pregnant women should be advised to avoid excessive energy intake and excessive weight gain during their pregnancy. Furthermore, data suggest that higher calcium and magnesium intake and a diet rich in fruit and vegetables may be beneficial for HDP. Adequate calcium and magnesium intake may be achieved by increasing intake of low-fat dairy and fruit and vegetables. There is a need for well-powered prospective cohort studies and intervention trials in a range of populations assessing nutrition prior to and during pregnancy, examining associations with the different subtypes of HDP.

## Additional files

Additional file 1:
**Additional tables and figures.**
Additional file 2:
**Difference in unadjusted nutrient intake between pre-eclampsia and/or gestational hypertension cases and non-cases.**
Additional file 3:
**Associations between nutrient intake and pre-eclampsia and/or gestational hypertension adjusted for confounding factors.**
Additional file 4:
**Difference in unadjusted food intake between pre-eclampsia and/or gestational hypertension cases and non-cases and associations between food consumption and pre-eclampsia and/or gestational hypertension adjusted for confounding factors.**

## Abbreviations

BMI: Body mass index
CI: Confidence interval
FFQ: Food frequency questionnaire
HDP: Hypertensive disorders of pregnancy
MoBa: Norwegian Mother and Child Cohort Study
OR: Odds ratio
RR: Relative risk
SD: Standard deviation
SE: Standard error
SEM: Standard error of the mean
WMD: Weighted mean difference
