# Supplementary material for: The association between dietary factors and gestational hypertension and pre-eclampsia: a systematic review and meta-analysis of observational studies
Source: BMC Med. 2014 Sep 22;12:157. doi: 10.1186/s12916-014-0157-7 (PMC4192458; doi:10.1186/s12916-014-0157-7)
Supplement: Additional file 1: — Additional tables and figures. [file 12916_2014_157_MOESM1_ESM.pdf]

Additional file 1: Additional Tables and Figures

Table S1A. Search strategy utilised for MEDLINE (years 1950 to May 2014)

Search #	Search term[a]	Results
1	exp Hypertension, Pregnancy-Induced/ OR gestational hypertension.tw. OR pregnancy induced hypertension.tw. OR pregnancy-induced hypertension.tw. OR exp Pre-Eclampsia/ OR preeclampsia.tw. OR pre-eclampsia.tw. OR hypertensi* disorder* of pregnancy.tw.	25,239
2	exp Diet/ OR diet*.tw. OR nutri*.tw. OR exp Food/ OR food.tw. OR exp Vitamins/ OR vitamin*.tw.	662,398
3	1 AND 2	1,523

[a] All restricted to English language and human populations

Table S1B. Search strategy utilised for EMBASE (years 1966 to May 2014)

Search #	Search term[a]	Results
1	pregnancy induced hypertension:ti,ab OR pregnancy-induced hypertension:ti,ab OR gestational hypertension:ti,ab OR preeclampsia/exp OR pre-eclampsia:ti,ab OR preeclampsia:ti,ab OR hypertensi* disorder* of pregnancy:ti,ab	19,033
2	diet/exp OR diet*:ti,ab OR nutrition/exp OR nutri*:ti,ab OR food/exp OR food:ti,ab OR vitamin/exp OR vitamin*:ti,ab	294,898
3	1 AND 2	720

[a] All restricted to English language and human populations

Table S2. Selected characteristics of cases and non-cases in studies included in systematic review of the association between dietary factors and hypertensive disorders of pregnancy

Study	Unit	Gestational hypertension cases	Preeclampsia cases	Non-cases	*P*-value
Maternal age, years - case-control studies					
Al MD, 1994	Mean (SEM)		27.9 (0.54)	28.5 (0.35)	ns
Atkinson JO, 1998	Mean (SD)		25.6 (6.4)	24.8 (7.9)	0.098
Duvekot EJ, 2002[a]	Median (SD)		28 (1)	28 (0.3)	
Frederick IO, 2005 [a]	Mean (SEM)		29.9 (0.5)	30.6 (0.3)	ns
Gulsen S, 2012	15-19y, *n* (%)		6 (6.5)	4 (2.6)	<0.0001
	20-34y, *n* (%)		60 (65.2)	136 (87.8)	
	>35y, *n* (%)		26 (28.3)	15 (9.7)	
Kazemian E, 2013	Mean (SD)	28.73 (6.04)		25.36 (4.84)	<0.001
Marcoux S, 1991	Mean (SD)	26.2 (4.3)	26.0 (4.8)	26.1 (4.2)	ns
Paknahad Z, 2008 [a]	Mean (SE)	26.5 (0.89)[c]	26.5 (0.89)[c]	24.6 (0.72)	0.1
Reyes L, 2012 [a]	Mean (SD)		26.45 (7.22)	26.71 (7.21)	ns
Richards DGD, 2014 [a]	Mean (SD)		24 (4.3)	24 (4.4)	0.7
Schiff E, 1996	Mean (SD)		21.5 (5.4)	20.1 (4.4)	ns
Sharbaf FR, 2013	Mean (SD)		28 (4.1)	27 (5)	0.53
Wei S-Q, 2009	Mean (SD)		29.0 (5.2)	29.1 (5.3)	0.55
Zhang C, 2002	Mean (SEM)		30.1 (0.6)	31.8 (0.3)	
Maternal age, years - cohort studies					
Clausen T, 2001	Mean (SD)		29.2 (4.9)	29.9 (4.5)	0.16
Geraldo Lopes Ramos J, 2006	Mean	26.1	Mild: 28.1<br>Severe: 23.3	25.2	
Goodarzi Khoigani M, 2012	Mean (SD)		26.48 (4.21)	25.60 (4.44)	0.33
Longo-Mbenza B, 2008	Mean (SD)	25.5 (7.2)[c]	25.5 (7.2)[c]	27.4 (6.4)	ns
Morris CD, 2001	<25y, *n* (%)	619 (83)	273 (84)	2744 (84)	ns
	25-30y, *n* (%)	91 (12)	34 (10)	335 (11)	
	>30y, *n* (%)	37 (5)	19 (6)	162 (5)	
Oken E, 2007	<20y, %	3	7	3	
	20-40y, %	94	87	93	
	>40y, %	3	7	4	
Olafsdottir AS, 2006	Mean (SD)	29 (6)	26 (4)	28 (5)	ns
Ortega RM, 1999	Mean (SD)	26.2 (3.4)		27.0 (3.9)	ns
Rumbold AR, 2005	Mean (SD)	28 (5)[c]	28 (5)[c]	28 (5)	ns
Tande DL, 2013	Mean (SE)	25.3 (0.72)[c]	25.3 (0.72)[c]	24.2 (0.62)	
Gestational age at delivery, weeks - case-control studies					
Al MD, 1994	Mean (SEM)		39.0 (0.3)	40.0 (0.08)	<0.005
Duvekot EJ, 2002	Median (SD)		35 (0.4)	39 (0.3)	
Gestational age at delivery, weeks - cohort studies					
Clausen T, 2001	Mean (SD)		37.8 (3.4)	39.5 (1.7)	<0.001
Rumbold AR, 2005	Mean (SD)	38 (1)[c]	38 (1)[c]	39 (1)	0.03
Tande DL, 2013	≤38 weeks, *n* (%)	8 (62.5)[c]	8 (62.5)[c]	18 (37.5)	
	≥39 weeks, *n* (%)	5 (38.5)[c]	5 (38.5)[c]	30 (62.5)	

Table S2. Continued

Study	Unit	Gestational hypertension cases	Preeclampsia cases	Non-cases	*P*-value
Body mass index, kg/m^2 - case-control studies					
Gulsen S, 2012	Mean (SE)		26.35 (0.55)	25.56 (0.91)	<0.53
Wei S-Q, 2009	Mean (SD)		23.9 (6.1)	22.6 (4.2)	0.13
Frederick IO, 2005	Mean (SEM)		27.1 (0.5)	22.9 (0.2)	<0.05
Kazemian E, 2013	Mean (SD)	28.97 (6.31)		23.70 (4.64)	<0.001
Richards DGD, 2014	Median (range)		28.6 (18.8-52.5)	28.4 (17.9-51.5)	0.9
Marcoux S, 1991	Mean (SD)	23.1 (4.4)	22.4 (3.8)	21.0 (2.8)	<0.05
Paknahad Z, 2008	Mean (SE)	25.65 (0.89)[c]	25.65 (0.89)[c]	23.6 (0.58)	0.05
Reyes L, 2012	Mean (SD)		27.92 (5.01)	26.68 (3.83)	<0.001
Zhang C, 2002	Mean (SEM)		26.0 (0.7)	22.5 (0.3)	
Body mass index, kg/m^2 - cohort studies					
Clausen T, 2001	Mean (SD)		23.7 (4.3)	22.9 (3.7)	0.04
Geraldo Lopes Ramos J, 2006	Mean	30.1	Mild: 30.0<br>Severe: 29.6	28.1	
Goodarzi Khoigani M, 2012	Mean (SD)		25.30 (5.29)	23.42 (3.84)	0.02
Longo-Mbenza B, 2008	Mean (SD)	22.9 (2.1)[c]	22.9 (2.1)[c]	22.7 (2)	ns
Morris CD, 2001	<19.8, *n* (%)	65 (9)	18 (5)	331 (10)	<0.0001
	19.8-25.9, *n* (%)	317 (42)	142 (44)	1794 (55)	
	26-35, *n* (%)	269 (36)	118 (36)	896 (28)	
	>35, *n* (%)	96 (13)	48 (15)	216 (7)	
Oken E, 2007	<25, %	48	37	67	
	25-30, %	29	27	21	
	>30, %	23	36	12	
Olafsdottir AS, 2006	Mean (SD)	28.7 (5.2)	26.4 (4.4)	24.4 (4.1)	<0.001 for GH
Ortega RM, 1999	Mean (SD)	21.6 (0.9)		21.9 (2.4)	ns
Rumbold AR, 2005	<25, *n* (%)	6 (19)[c]	6 (19)[c]	102 (55)	<0.0001
	25-30, *n* (%)	10 (31)[c]	10 (31)[c]	50 (27)	
	>30, *n* (%)	16 (50)[c]	16 (50)[c]	32 (17)	
Nulliparous - case-control studies					
Al MD, 1994[b]	*n* (%)		135 (86.5)	45 (86.5)	ns
Atkinson JO, 1998	*n* (%)		96 (53.3)	76 (39.2)	0.008
Frederick IO, 2005[b]	*n* (%)		123 (71.5)	182 (53.7)	<0.05
Kesmodel U, 1997	*n* (%)	77 (62.6)	24 (72.7)	109 (56.2)	
Zhang C, 2002	*n* (%)		49 (45.0)	91 (35.1)	
Nulliparous - cohort studies					
Clausen T, 2001	*n* (%)		63 (74.1)	1560 (51.2)	<0.001
Oken E, 2007	%	66	73	47	
Olafsdottir AS, 2006	%	57	79	47	<0.05 for PE
Birth weight, grams - case-control studies					
Al MD, 1994	Mean (SEM)		2941 (99.5)	3290 (30.2)	<0.001
Sharbaf FR, 2013	Mean (SD)		2860 (1.0)	3316 (5.1)	0.00
Wei S-Q, 2009	Mean (SD)		2126 (898)	3224 (657)	0.0001

Table S2. Continued

Study	Unit	Gestational hypertension cases	Preeclampsia cases	Non-cases	*P*-value
Birth weight, grams - cohort studies					
Clausen T, 2001	Mean (SD)		3072 (932)	3598 (535)	<0.001
Geraldo Lopes Ramos J, 2006	Mean	3353	Mild: 2013<br>Severe: 2106	3158	
Longo-Mbenza B, 2008	Mean (SD)	2341 (657)[c]	2341 (657)[c]	2817 (543)	<0.05
Olafsdottir AS, 2006	Mean (SD)	3888 (562)	3602 (526)	3779 (479)	ns
Rumbold AR, 2005	Mean (SD)	3585 (598)[c]	3585 (598)[c]	3503 (482)	ns

GH, gestational hypertension; ns, not significant; PE, preeclampsia

[a] cases and controls matched for maternal age

[b] cases and controls matched for parity

[c] for gestational hypertension and/or preeclampsia

Table S3. Overview of diagnostic criteria for gestational hypertension and preeclampsia used in studies on dietary factors and hypertensive disorders of pregnancy

Study	Diagnostic criteria
**Gestational hypertension**	
Geraldo Lopes Ramos J, 2006	Blood pressure (>140/90mmHg)[a,b]
Kazemian E, 2013	Blood pressure ≥140/90mmHg on ≥2 occasions[a,b]
Kesmodel U, 1997	Diastolic blood pressure >90mmHg
Marcoux S, 1991	Diastolic blood pressure ≥90mmHg[a,b]
Morris CD, 2001	Diastolic blood pressure ≥90mmHg on ≥2 occasions 4-168h apart
Oken E, 2007	Blood pressure >140/90mmHg on ≥2 occasions[b]
Olafsdottir AS, 2006	Blood pressure ≥140/90mmHg[a,b]
Ortega RM, 1999	Blood pressure >140/90 mmHg on≥2 occasions 1 week apart[a,b]
Rifas-Shiman SL, 2009	Blood pressure >140/90mmHg on ≥2 occasions [a,b]
Rumbold AR, 2005	Blood pressure ≥140/90mmHg on 2 occasions ≥4h apart [a,b]
Saftlas AF, 2010	Blood pressure ≥140/90mmHg on ≥2 occasions ≥6h apart [a,b]
Timmermans S, 2011	Blood pressure ≥140/90mmHg on ≥2 occasions [a,b]
**Preeclampsia**	
Al MD, 1994	Blood pressure (increase during pregnancy of ≥25mmHg, or two consecutive measurements of ≥90mmHg or one of ≥110mmHg) and proteinuria (≥300mg/24h)[a,b]
Borgen I, 2012	Blood pressure (>140/90mmHg) and proteinuria (>1+ on a dipstick ≥2 occasions)[a,c]
Brantsæter AL, 2009	Blood pressure (>140/90mmHg) and proteinuria (>1+ on a dipstick ≥2 occasions)[a,c]
Brantsæter AL, 2011	Blood pressure (>140/90mmHg) and proteinuria (>1+ on a dipstick ≥2 occasions)[a,c]<br>Severe preeclampsia: blood pressure (>160/110mmHg) and proteinuria (>1+ on a dipstick ≥2 occasions)[a,c]
Chavarro JE, 2011	Blood pressure (≥140/90mmHg) and proteinuria (≥300mg/24h or ≥1+ on a dipstick)[a]
Clausen T, 2001	Blood pressure (≥140/90mmHg or increase in diastolic pressure of ≥15mmHg above first trimester values on 2 occasions ≥6h apart) and proteinuria (300mg/24h or ≥+1 on a dipstick in ≥2 random urine samples taken ≥6h apart)[a]<br>Early-onset preeclampsia: onset <37 weeks' gestation<br>Late-onset preeclampsia: onset ≥37 weeks' gestation
Duvekot EJ, 2002	Blood pressure (rise of ≥30mmHg systolic or 15mmHg diastolic above first 20 weeks of pregnancy values) and proteinuria (≥100mg/dl or ≥2+ on a dipstick)[a,b]
Frederick IO, 2005	Blood pressure (sustained rice of 30mmHg diastolic or 15mmHg diastolic above first trimester values, or persistent (≥6 hours) blood pressure >140/90mmHg) and proteinuria (≥30mg/dl or 1+ on a dipstick on ≥2 random samples collected ≥4h apart)[a]

Table S3. Continued

Study	Diagnostic criteria
Goodarzi Khoigani M, 2012	Blood pressure (≥140/90mmHg) and proteinuria (≥300mg/24h)[a]
Geraldo Lopes Ramos J, 2006	Blood pressure (>140/90mmHg) and proteinuria (≥300mg/24h or 30mg/dL or ≥1+ on a dipstick)[a,b]<br>Severe preeclampsia: blood pressure (>180/110mmHg) and proteinuria (≥3000mg/24h or ≥3+ on a dipstick)[a,b]
Gulsen S, 2012	Blood pressure (>140/90mmHg) and proteinuria (>100mg/dl or >300mg/24h)[a,b]
Haugen M, 2009	Blood pressure (>140/90mmHg) and proteinuria (>1+ on a dipstick on ≥2 occasions)[a,c]
Kesmodel U, 1997	Diastolic blood pressure >90mmHg and proteinuria (>0.3g/l or >+ on a dipstick)
Klemmensen AK, 2009	Blood pressure (≥140/90mmHg on 2 occasions ≥6h apart) and proteinuria (≥300mg/24h or ≥1+ on a dipstick on 2 occasions ≥4h apart)[a,b]<br>Severe preeclampsia: blood pressure (≥160/110mmHg on 2 occasions ≥6h apart) and proteinuria (≥5000mg/24h or ≥3+ on a dipstick on 2 occasions ≥4h apart) or oliguria, cerebral or visual disturbances, pulmonary edema or cyanosis, epigastric or upper right-quadrant pain, impaired liver function, thrombocytopenia, or fetal growth restriction [a,b]
Marcoux S, 1991	Blood pressure (diastolic ≥90mmHg) and proteinuria (≥300mg/24h or ≥1g/L in 2 urine samples)[a,b]
Morris CD, 2001	Blood pressure (diastolic ≥90mmHg on ≥2 occasions 4-168h apart) and proteinuria (≥300mg/24h, ≥1+ on a dipstick in 2 random urine samples taken 4-168h apart, or ≥2+ on a dipstick in a single sample or protein/creatinine ratio ≥0.35)
Oken E, 2007	Blood pressure (>140/90mmHg on ≥2 occasions) and proteinuria (1+ on a dipstick in ≥2 urine samples or >2+ on a dipstick in a single sample 4h-7days apart)[a,c]
Olafsdottir AS, 2006	Blood pressure (≥140/90mmHg) and proteinuria (≥300mg/24h, ≥1+ on a dipstick)[a,b]
Qiu C, 2008	Blood pressure (≥140/90mmHg taken ≥6h apart or a sustained 30mmHg systolic or 15mmHg diastolic rise above first trimester values) and proteinuria (≥30mg/dl on ≥2 random samples taken ≥4h apart)[a,b]
Reyes L, 2012	Blood pressure (≥140/90mmHg) and proteinuria (≥300mg/24h or ≥1+ on a dipstick in ≥2 random urine samples taken ≥4-6h apart)[a,b]
Richards DGD, 2014	Blood pressure (≥160/110mmHg on 1 occasion or ≥140/90mmHg on 2 occasions 4h apart) and proteinuria (≥300mg/24h, ≥2+ on a dipstick)[a, b]
Richardson BE, 1995	Blood pressure (≥140/90mmHg ≥2 occasions ≥6h apart) and albuminuria (1+ on a dipstick) and/or edema[b]
Rifas-Shiman SL, 2009	Blood pressure (>140/90mmHg on ≥2 occasions) and proteinuria (1+ on a dipstick in ≥2 urine samples or >2+ on a dipstick in a single sample 4h-7days apart)[a,c]

Table S3. Continued

Study	Diagnostic criteria
Rumbold AR, 2005	Blood pressure (≥140/90mmHg on 2 occasions ≥4h apart) and one or more of the following: proteinuria (≥300mg/24h or protein/creatinine ratio ≥30mg/mmol,) renal insufficiency (serum/plasma creatinine ≥0.09mmol/L or oliguria), liver disease, neurological problems, haematological disturbances or fetal growth restriction)[a,b]
Saftlas AF, 2010	Blood pressure (≥140/90mmHg on ≥2 occasions ≥6h apart) and proteinuria (≥300mg/24h or ≥1+ on a dipstick on ≥2 occasions)[a,b]
Schiff E, 1996	Blood pressure (≥145/95mmHg on ≥2 occasions 6h apart) and proteinuria (≥500mg/24h or ≥2+ on a dipstick)[a,b]
Sharbaf FR, 2013	Blood pressure (≥140/90mmHg on ≥2 occasions ≥4h apart) and proteinuria (≥300mg/24h or ≥1+ on a dipstick)[a,b]
Skajaa K, 1991	Blood pressure (≥140/95mmHg) and proteinuria (≥0.3g/l)[a,b]
Timmermans S, 2011	Blood pressure (≥140/90mmHg on ≥2 occasions) and proteinuria (≥300mg/24h, ≥1+ on a dipstick on 1 occasion, or ≥2+ urine dipstick on ≥2 occasion)[a,b]
Triche EW, 2008	Blood pressure (≥140/90mmHg on ≥2 occasions ≥6h apart) and proteinuria (≥30mg/dL, or ≥300mg/24h, or 1+ on a dipstick on ≥2 samples collected ≥4h apart or ≥2+ on a dipstick on ≥1 sample near end of pregnancy, or 1+ on a dipstick on ≥1 catheterized dipstick during delivery hospitalization)[a,b]
Wei S-Q, 2009	Blood pressure (≥140/90mmHg on ≥2 occasions ≥4h apart) and proteinuria (≥300 mg/24h or ≥1+ on a dipstick)[a,b]<br>Severe preeclampsia: blood pressure (≥160/110mmHg on ≥2 occasions ≥6h apart) and proteinuria (≥5000mg/24h or ≥3+ on a dipstick) or oliguria, pulmonary edema or cyanosis, impairment of liver function, visual or cerebral disturbances, decreased platelet count, or intrauterine growth restriction
Zhang C, 2002	Blood pressure (sustained rice of 30mmHg diastolic or 15mmHg diastolic above first trimester values, or persistent (≥6h) blood pressure >140/90mmHg) and proteinuria (≥30mg/dl or 1+ on a dipstick on ≥2 random samples collected ≥4h apart)[a,b]
**Gestational hypertension or preeclampsia**	
Longa-Mbenza B, 2008	Blood pressure (sustained rice of 30mmHg diastolic or 15mmHg diastolic above first trimester values, or persistent (≥6h) blood pressure >140/90mmHg) with or without proteinuria (≥30mg/dl or 1+ on a dipstick on ≥2 random samples collected ≥4h apart)[a,b]
Paknahad Z, 2008	Blood pressure >140/90mmHg with or without proteinuria (>300mg/day) in the absence of urinary tract infection[a,b]
Tande DL, 2013	Blood pressure ≥140/90mmHg on 2 occasions ≥6h apart with or without proteinuria (>300mg/24h or 1+ on a dipstick)[a,b]

[a] Age at onset >20 weeks' gestation
[b] Women with pre-existing or chronic hypertension were excluded from analysis
[c] Women with chronic hypertension were excluded, but not if they developed proteinuria

Table S4A. Quality assessment of case-control studies on dietary factors and hypertensive disorders of pregnancy

	Selection				Comparability	Exposure			
Study	Case definition	Case representativeness	Control selection	Control definition	Comparability of cases and controls on bases of design or analysis	Exposure ascertainment	Similar ascertainment method for cases and controls	Non-response rate	Total score
Al MD, 1994	A★	A★	B	A★	-	D	A★	B	4
Atkinson JO, 1998	A★	A★	B	A★	-	D	A★	B	4
Duvekot EJ, 2002	A★	A★	B	A★	A★ B★	D	A★	B	6
Frederick IO, 2005	A★	A★	B	A★	A★ B★	B★	A★	A★	8
Gulsen S, 2012	A★	A★	B	A★	-	D	A★	B	4
Kazemian E, 2013	A★	A★	B	A★	A★ B★	B★	A★	B	7
Kesmodel U, 1997	A★	A★	A★	A★	-	D	A★	B	5
Marcoux S, 1991	A★	A★	B	B	-	D	A★	B	3
Paknahad Z, 2008	A★	A★	B	A★	-	B★	A★	B	5
Reyes L, 2012	A★	A★	A★	A★	-	B★	A★	B	6
Richards DGD, 2014	A★	A★	B	A★	-	D	A★	A★	5
Schiff E, 1996	A★	A★	A★	A★	-	D	A★	B	5
Sharbaf FR, 2013	A★	A★	B	A★	-	D	A★	B	4
Wei S-Q, 2009	A★	A★	B	A★	A★ B★	D	A★	B	6
Zhang C, 2002	A★	A★	B	A★	A★ B★	B★	A★	A★	8

Table S4B. Quality assessment of cohort studies on dietary factors and hypertensive disorders of pregnancy

	Selection				Compara-bility	Outcome			
Study	Representativeness of exposed cohort	Selection of non-exposed cohort	Ascertainment of exposure	Outcome not present at start of study	Comparability of cases and controls on bases of design or analysis	Outcome assessment	Adequate during of follow-up	Adequacy of follow-up rate	Total score
Borgen I, 2012	B★	A★	B★	A★	A★ B★	B★	A★	B★	9
Brantsæter AL, 2009	B★	A★	B★	A★	A★ B★	B★	A★	B★	9
Brantsæter AL, 2011	B★	A★	B★	A★	A★ B★	B★	A★	B★	9
Chavarro JE, 2011	B★	A★	B★	B	-	B★	A★	B★	6
Clausen T, 2001	B★	A★	B★	B	-	A★	A★	B★	6
Geraldo Lopes Ramos J, 2006	D	A★	B★	B	-	A★	A★	B★	5
Goodarzi Khoigani M, 2012	B★	A★	B★	A★	-	B★	A★	B★	7
Haugen M, 2009	B★	A★	B★	A★	A★ B★	B★	A★	B★	9
Klemmensen AK, 2009	B★	A★	B★	B	A★ B★	B★	A★	B★	8
Longo-Mbenza B, 2008	C	A★	C	A★	-	A★	A★	B★	5
Morris CD, 2001	C	A★	B★	A★	-	A★	A★	B★	6
Oken E, 2007	C	A★	B★	A★	A★ B★	B★	A★	B★	8
Olafsdottir AS, 2006	D	A★	B★	A★	-	B★	A★	B★	6
Ortega RM, 1999	D	A★	B★	B	-	A★	A★	B★	5
Qiu C, 2008	D	A★	B★	A★	A★ B★	A★	A★	D	7

Table S4B. Continued

	Selection				Comparability	Outcome			
Study	Representativeness of exposed cohort	Selection of non-exposed cohort	Ascertainment of exposure	Outcome not present at start of study	Comparability of cases and controls on bases of design or analysis	Outcome assessment	Adequate during of follow-up	Adequacy of follow-up rate	Total score
Richardson BE, 1995	D	A★	B★	B	-	B★	A★	D	4
Rifas-Shiman SL, 2009	D	A★	B★	B	A★ B★	A★	A★	B★	7
Rumbold AR, 2005	C	A★	B★	B	A★ B★	A★	A★	D	6
Saftlas AF, 2010	D	A★	B★	A★	A★ B★	B★	A★	B★	8
Skajaa K, 1991	D	A★	B★	B	A★ B★	B★	A★	B★	7
Tande DL, 2013	C	A★	C	A★	-	B★	A★	B★	5
Timmermans S, 2011	B★	A★	B★	A★	A★ B★	B★	A★	D	8
Triche EW, 2008	B★	A★	B★	A★	A★ B★	B★	A★	B★	9

Newcastle – Ottawa quality assessment scale

Case-control studies

Note: A study can be awarded a maximum of one star for each numbered item within the Selection and Exposure categories. A maximum of two stars can be given for Comparability.

Selection

1) Is the case definition adequate?
a) yes, with independent validation ★
b) yes, e.g. record linkage or based on self-reports
c) no description

2) Representativeness of the cases
a) consecutive or obviously representative series of cases ★
b) potential for selection biases or not stated

3) Selection of Controls
a) community controls ★
b) hospital controls
c) no description

4) Definition of Controls
a) no history of disease (endpoint) ★
b) no description of source

Comparability

1) Comparability of cases and controls on the basis of the design or analysis
a) study controls for *maternal age, hypertension prior to pregnancy and parity* ★
b) study controls for any additional factor ★ (*e.g. body mass index, smoking, socioeconomic factors, family history of hypertension or HDP, physical activity, dietary factors*)

Exposure

1) Ascertainment of exposure
a) secure record (*e.g. hospital record*) ★
b) structured interview/*dietary recall/diet history/validated food frequency questionnaire* where blind to case/control status ★
c) interview/*dietary recall/diet history/validated food frequency questionnaire* not blinded to case/control status
d) written self-report or medical record only (*e.g. non-validated food frequency questionnaire*)
e) no description

2) Same method of ascertainment for cases and controls
a) yes ★
b) no

3) Non-Response rate
a) same rate for both groups ★
b) non respondents described
c) rate different and no designation

Cohort studies

Note: A study can be awarded a maximum of one star for each numbered item within the Selection and Outcome categories. A maximum of two stars can be given for Comparability

Selection

1) Representativeness of the exposed cohort
a) truly representative of the average *reproductive-aged women* in the community ★
b) somewhat representative of the average *reproductive-aged women* in the community ★
c) selected group of users *e.g. only certain socio-economic groups/areas*
d) no description of the derivation of the cohort

2) Selection of the non-exposed cohort
a) drawn from the same community as the exposed cohort ★
b) drawn from a different source
c) no description of the derivation of the non-exposed cohort

3) Ascertainment of exposure
a) secure record (*e.g. hospital record*) ★
b) structured interview/*dietary recall/diet history/validated food frequency questionnaire* ★
c) written self-report (*e.g. non-validated food frequency questionnaire*)
d) no description

4) Demonstration that outcome of interest was not present at start of study
a) yes ★
b) no

Comparability

1) Comparability of cohorts on the basis of the design or analysis
a) study controls for *maternal age, hypertension prior to pregnancy and parity* ★
b) study controls for any additional factor ★ (*e.g. body mass index, smoking, socioeconomic factors, family history of hypertension or HDP, physical activity, dietary factors*)

Outcome

1) Assessment of outcome
a) independent blind assessment ★
b) record linkage ★
c) self-report
d) no description

2) Was follow-up long enough for outcomes to occur
a) yes, *if followed-up until delivery* ★
b) no

3) Adequacy of follow up of cohorts
a) complete follow up - all subjects accounted for ★
b) subjects lost to follow up unlikely to introduce bias - small number lost *≤20%* follow up, or description provided of those lost ★
c) follow up rate <*80%* or no description of those lost
d) no statement

Table S5. Overview of confounding factors used in multivariable analysis in studies on dietary factors and hypertensive disorders of pregnancy

	Socio-demographic factors								Reproductive factors			Lifestyle factors						Dietary factors		
Study	Maternal age	Ethnicity	Socio-economic status[a]	Family history of HT	HT prior to pregnancy[b]	Previous HDP	Blood pressure in early pregnancy	Season of childbirth	Gestational age	Parity[c]	Pregnancy interval	Pre-pregnancy BMI	BMI	Height	Gestational weight gain	Smoking	Physical activity	Total energy intake	Dietary supplement use	Other nutrients/foods/pattern
Atkinson JO, 1998	✓				NA															
Borgen I, 2012	✓		✓		NA					NA		✓		✓		✓	✓	✓		✓
Brantsæter AL, 2009	✓		✓		✓					NA		✓		✓		✓		✓	✓	✓
Brantsæter AL, 2011	✓		✓		NA					NA		✓		✓		✓		✓		✓
Chavarro JE, 2011	✓		✓							✓		✓		✓		✓		✓		
Clausen T, 2001	✓						✓			✓			✓			✓		✓		✓
Duvekot EJ, 2002	✓			✓	NA					NA			✓			✓				
Frederick IO, 2005	✓		✓							✓		✓						✓		
Haugen M, 2009	✓		✓		NA			✓		NA		✓		✓		✓				
Kazemian E, 2013	✓			✓	NA	✓				✓	✓	✓			✓			✓		
Kesmodel U, 1997			✓							✓		✓		✓		✓				✓
Klemmensen AK, 2009	✓		✓		NA					✓		✓		✓		✓	✓			✓
Marcoux S, 1991	✓		✓		NA		✓						✓			✓	✓			
Morris CD, 2001	✓	✓							✓	NA			✓			✓		✓		

Table S5. Continued

Study	Socio-demographic factors								Reproductive factors			Lifestyle factors						Dietary factors		
	Maternal age	Ethnicity	Socio-economic status[a]	Family history of HT	HT prior to pregnancy[b]	Previous HDP	Blood pressure in early pregnancy	Season of childbirth	Gestational age	Parity[c]	Pregnancy interval	Pre-pregnancy BMI	BMI	Height	Gestational weight gain	Smoking	Physical activity	Total energy intake	Dietary supplement use	Other nutrients/foods/pattern
Oken E, 2007	✓	✓	✓		✓					✓		✓				✓		✓		
Qiu C, 2008	✓	✓			NA					✓		✓				✓		✓		✓
Reyes L, 2012	✓		✓		NA								✓							
Richardson BE, 1995					NA	✓				✓			✓		✓					
Rifas-Shiman SL, 2009	✓	✓	✓		NA					✓			✓							
Rumbold AR, 2005	✓	✓	✓	✓	NA	✓				✓			✓			✓				
Saftlas AF, 2010					NA					✓			✓							
Skajaa K, 1991	✓				NA				✓	✓		✓		✓	✓	✓				
Timmermans S, 2011	✓	NA	✓		NA					✓			✓			✓				✓
Triche EW, 2008	✓	✓	✓		NA					✓			✓			✓				
Wei S-Q, 2009	✓		✓		NA					NA			✓			✓				
Zhang C, 2002	✓	✓	✓		NA					✓		✓				✓		✓		

BMI, body mass index; HDP, hypertensive disorders of pregnancy; HT, hypertension; NA, not applicable

[a] Education, occupation or income

[b] NA if women with chronic hypertension were excluded from the analysis

[c] NA if the study includes nulliparous women only

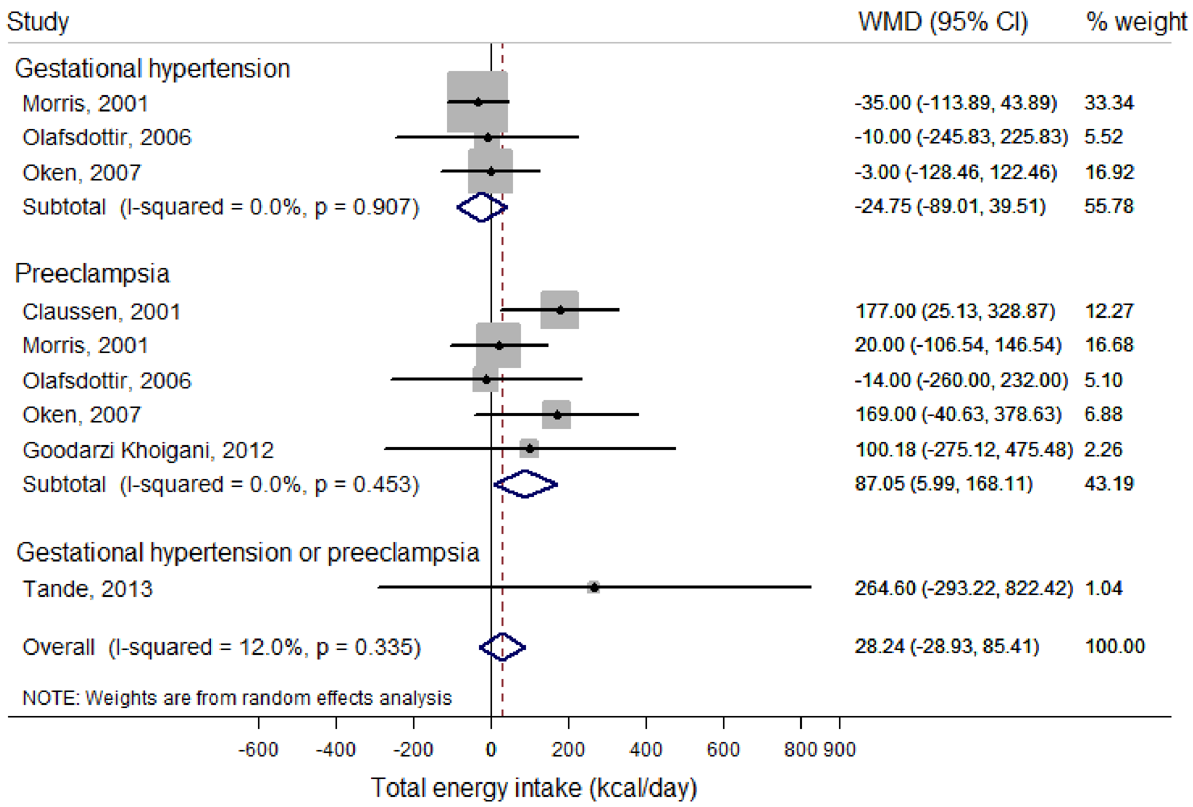

Figure S1. Difference of unadjusted total energy intake (kcal/day) between cases (gestational hypertension, preeclampsia and gestational hypertension or preeclampsia) and non-cases (reference) reported in cohort studies.

Meta-analysis of 9 studies from 6 articles with data from 10,302 pregnant women and 896 gestational hypertension, 512 preeclampsia, and 13 gestational hypertension or preeclampsia cases). For each study, the centre of each square indicates the weighted mean difference (WMD) and the horizontal line indicates the 95% confidence interval; the area of the square is proportional to the weight that the individual study contributes to the overall pooled mean difference; the diamonds are pooled mean differences (for each outcome and overall).
