# Supplementary material for: The association between dietary factors and gestational hypertension and pre-eclampsia: a systematic review and meta-analysis of observational studies
Source: BMC Med. 2014 Sep 22;12:157. doi: 10.1186/s12916-014-0157-7 (PMC4192458; doi:10.1186/s12916-014-0157-7)
Supplement: Additional file 2: — Difference in unadjusted nutrient intake between pre-eclampsia and/or gestational hypertension cases and non-cases. [file 12916_2014_157_MOESM2_ESM.doc]

**Additional file 2** Difference in unadjusted nutrient intake between pre-eclampsia and/or gestational hypertension cases and non-cases

|  | Pre-eclampsia | | | | | | | | | | | | | | | | | | Gestational hypertension | | | | | | | | HDP | |
| --- | --- | --- | --- | --- | --- | --- | --- | --- | --- | --- | --- | --- | --- | --- | --- | --- | --- | --- | --- | --- | --- | --- | --- | --- | --- | --- | --- | --- |
|  | Cohort studies | | | | | | | | | | Case–control studies | | | | | | | | Cohort studies | | | | Case–control studies | | | | Coh | C-c |
|  | Brantsæter et al., 2011 [20] | Goodarzi Khoigani et al., 2012 [22] | Haugen M, 2009 [24] | Klemmensen et al., 2009 [26] | Borgen et al, 2012 [37] | Clausen et al., 2001 [40] | Morris et al., 2001 [42] | Oken et al., 2007 [43] | Olafsdottir et al., 2006 [44] | Skajaa et al., 1991 [49] | Frederick et al., 2005 [21] | Kesmodel et al., 1997 [25] | Marcoux et al., 1991 [27] | Geraldo Lopes Ramos et al., 2006 [35] | Al et al., 1994 [52] | Reyes et al., 2012 [56] | Schiff et al., 1996 [58] | Zhang et al., 2002 [60] | Morris et al., 2001 [42] | Oken et al., 2007 [43] | Olafsdottir et al., 2006 [44] | Ortega et al., 1999 [45] | Kesmodel et al., 1997 [25] | Marcoux et al., 1991 [27] | Geraldo Lopes Ramos et al., 2006 [35] | Kazemian et al., 2013 [55] | Tande et al., 2013 [50] | Paknahad et al., 2008 [28] |
| Energy and macronutrients |  |  |  |  |  |  |  |  |  |  |  |  |  |  |  |  |  |  |  |  |  |  |  |  |  |  |  |  |
| Total energy | x | x | x |  | x | ↑ | x | x | x | x | x |  |  |  | x | ↑ |  |  | x | x | x |  |  |  |  | ↑ | x | x |
| Protein | ↓ | x |  |  |  | ↓ | x |  | x | x | x |  |  |  | x | x |  |  | x |  | x |  |  |  |  | x | x | ↓ |
| Total carbohydrate |  | ↓^d^ |  |  |  |  | x |  | x | x | ↓ |  |  |  | x | ↑ |  |  | x |  | x |  |  |  |  | ↓ | x |  |
| Sucrose |  |  |  |  |  | ↑ |  |  |  |  |  |  |  |  |  |  |  |  |  |  |  |  |  |  |  |  |  |  |
| Non-sucrose carbohydrate |  |  |  |  |  | x |  |  |  |  |  |  |  |  |  |  |  |  |  |  |  |  |  |  |  |  |  |  |
| Mono- and di-saccharides |  |  |  |  | x |  |  |  |  |  |  |  |  |  |  |  |  |  |  |  |  |  |  |  |  |  |  |  |
| Added sugar |  |  |  |  | ↑ |  |  |  | ↓ |  |  |  |  |  |  |  |  |  |  |  |  |  |  |  |  |  |  |  |
| Total fiber | ↓ | ↓^d^ |  |  |  |  | x |  |  | x | ↓ |  |  |  |  | x |  |  | x |  |  |  |  |  |  |  | x |  |
| Water-soluble fiber |  |  |  |  |  |  |  |  |  |  | ↓ |  |  |  |  |  |  |  |  |  |  |  |  |  |  |  |  |  |
| Water-insoluble fiber |  |  |  |  |  |  |  |  |  |  | x |  |  |  |  |  |  |  |  |  |  |  |  |  |  |  |  |  |
| Total fat | x |  |  |  |  | x | x |  | x | x | x |  |  |  | x | x |  |  | x |  | x |  |  |  |  | ↑ | x |  |
| Saturated fat | x | ↑^c^ |  |  |  | x | x |  | x |  |  |  |  |  | x | x |  |  | x |  | x |  |  |  |  | ↑ | x |  |
| Trans fat |  |  |  |  |  |  |  | x |  |  |  |  |  |  |  |  |  |  |  | x |  |  |  |  |  |  |  |  |
| Monounsaturated fat |  | x |  |  |  | x |  |  | x |  |  |  |  |  | x | x |  |  |  |  | x |  |  |  |  | ↑ |  |  |
| Polyunsaturated fat |  | x |  |  |  | x | x |  | x |  |  |  |  |  | x |  |  |  | x |  | x |  |  |  |  | ↑ |  |  |
| Cholesterol |  | x |  |  |  |  | x |  |  |  |  |  |  |  |  |  |  |  | x |  |  |  |  |  |  |  |  |  |
| n-3 fatty acids |  |  | ↓^a^ |  |  | x |  | ↓ | x |  |  | x |  |  |  |  |  |  |  | x | x |  | x |  |  |  |  |  |
| n-6 fatty acids |  |  |  |  |  | x |  | x | x |  |  |  |  |  |  |  |  |  |  | x | x |  |  |  |  |  |  |  |
| Micronutrients and minerals |  |  |  |  |  |  |  |  |  |  |  |  |  |  |  |  |  |  |  |  |  |  |  |  |  |  |  |  |
| Vitamin A |  | x |  |  |  |  | x |  |  |  |  |  |  |  |  |  |  |  | x |  |  |  |  |  |  | x |  |  |
| Vitamin B |  | x |  |  |  |  | x |  |  |  |  |  |  |  |  | x |  |  | x |  |  |  |  |  |  |  |  |  |
| Vitamin C |  | ↓^d^ |  | x |  |  | x | ↓ |  |  |  |  |  |  |  | x |  | ↓ | x | ↑ |  |  |  |  |  | ↓ |  | x |
| Vitamin D | x | x | ↓^a^ |  |  |  |  | x | x |  |  |  |  |  |  |  |  |  |  | ↑ | x |  |  |  |  | x |  |  |
| Vitamin E |  | ↓^d^ |  | x |  |  | x | ↓ | x |  |  |  |  |  |  |  | ↑^a^ |  | x | ↑ | x |  |  |  |  | ↑ |  | x |
| Vitamin K |  |  |  |  |  |  |  |  |  |  |  |  |  |  |  |  |  |  |  |  |  |  |  |  |  | ↓ |  |  |
| Calcium | x | x |  |  |  |  | x | x |  |  | x | x | x | ↓^b^ |  | x |  |  | x | x |  | ↓ | x | ↓ | x | x | x | ↓ |
| Iron |  | x |  |  |  |  | x |  |  |  |  |  |  |  |  | x |  |  | x |  |  |  |  |  |  | x | x |  |
| Potassium |  | x |  |  |  |  | x |  |  |  | x |  |  |  |  | x |  |  | x |  |  |  |  |  |  | ↓ | x |  |
| Sodium |  | x |  |  |  |  | x |  |  |  |  |  |  |  |  | x |  |  | x |  |  |  |  |  |  |  | x |  |
| Selenium |  | x |  |  |  |  |  |  |  |  |  |  |  |  |  |  |  |  |  |  |  |  |  |  |  | x |  |  |
| Phosphorus |  | x |  |  |  |  | x |  |  |  |  |  |  |  |  |  |  |  | x |  |  |  |  |  |  |  |  |  |
| Magnesium |  | x |  |  |  |  | x | ↓ |  | x | x |  |  |  |  |  |  |  | x | x |  |  |  |  |  | ↓ |  |  |
| Zinc |  | x |  |  |  |  | x |  |  |  |  |  |  |  |  |  |  |  | x |  |  |  |  |  |  | x | x | ↓ |
| Copper |  | x |  |  |  |  |  |  |  |  |  |  |  |  |  |  |  |  |  |  |  |  |  |  |  | x | x |  |
| Folate |  | x |  |  |  |  | x | ↓ |  |  |  |  |  |  |  | x |  |  | x | x |  |  |  |  |  |  | x |  |
| Niacin |  |  |  |  |  |  | x |  |  |  |  |  |  |  |  | x |  |  | x |  |  |  |  |  |  |  |  |  |
| Riboflavin |  |  |  |  |  |  |  |  |  |  |  |  |  |  |  | x |  |  |  |  |  |  |  |  |  |  |  | ↓ |
| Thiamin |  |  |  |  |  |  |  |  |  |  |  |  |  |  |  | x |  |  |  |  |  |  |  |  |  |  |  |  |
| Beta-carotene |  | x |  |  |  |  |  |  |  |  |  |  |  |  |  | x |  |  |  |  |  |  |  |  |  |  |  |  |
| Manganese |  | ↓^d^ |  |  |  |  |  |  |  |  |  |  |  |  |  |  |  |  |  |  |  |  |  |  |  | x |  |  |
| Retinol |  |  |  |  |  |  |  |  | x |  |  |  |  |  |  | x |  |  |  |  | x |  |  |  |  |  |  |  |

C-c, Case–control study; Coh, Cohort study; HDP, hypertensive disorders of pregnancy including both pre-eclampsia and gestational hypertension; ↓, significantly lower intake for pre-eclampsia and/or gestational hypertension cases compared with non-cases; ↑, significantly higher intake for pre-eclampsia and/or gestational hypertension cases compared with non-cases; x, no statistically significant difference between intake for pre-eclampsia and/or gestational hypertension cases and non-cases.

^a^ Significant difference for diet and supplements, not from diet only.

^b^ Severe pre-eclampsia (definition see Additional file 1: Table S3).

^c^ First trimester intake.

^d^ Third trimester intake.
