# Supplementary material for: The association between dietary factors and gestational hypertension and pre-eclampsia: a systematic review and meta-analysis of observational studies
Source: BMC Med. 2014 Sep 22;12:157. doi: 10.1186/s12916-014-0157-7 (PMC4192458; doi:10.1186/s12916-014-0157-7)
Supplement: Additional file 3: — Associations between nutrient intake and pre-eclampsia and/or gestational hypertension adjusted for confounding factors. [file 12916_2014_157_MOESM3_ESM.doc]

**Additional file 3** Associations between nutrient intake and pre-eclampsia and/or gestational hypertension adjusted for confounding factors

|  | Pre-eclampsia | | | | | | | | | | | | | | Gestational hypertension | | | | | HDP cohort studies |
| --- | --- | --- | --- | --- | --- | --- | --- | --- | --- | --- | --- | --- | --- | --- | --- | --- | --- | --- | --- | --- |
|  | Cohort studies | | | | | | | | | Case–control studies | | | | | Cohort studies | | Case–control studies | | |  |
|  | Haugen et al., 2009 [24] | Klemmensen et al., 2009 [26] | Qiu et al., 2008 [29] | Borgen et al., 2012 [37] | Chavarro et al., 2011 [39] | Clausen et al., 2001 [40] | Morris et al., 2001 [42] | Oken et al., 2007 [43] | Skajaa et al., 1991 [49] | Frederick et al., 2005 [21] | Kesmodel et al., 1997 [25] | Marcoux et al., 1991 [27] | Reyes et al., 2012 [56] | Zhang et al., 2002 [60] | Morris et al., 2001 [42] | Oken et al., 200 7[43] | Kesmodel et al., 1997 [25] | Marcoux et al., 1991 [27] | Kazemian et al., 2013 [55] | Rumbold et al., 2005 [48] |
| Energy and macronutrients |  |  |  |  |  |  |  |  |  |  |  |  |  |  |  |  |  |  |  |  |
| Total energy |  |  |  |  |  | ↑ |  |  |  |  |  |  |  |  |  |  |  |  | ↑ |  |
| Protein |  |  |  |  |  | x | x |  |  |  |  |  |  |  | x |  |  |  | x |  |
| Total carbohydrate |  |  |  |  |  |  |  |  |  |  |  |  | ↑ |  |  |  |  |  | x |  |
| Sucrose |  |  |  |  |  | x |  |  |  |  |  |  |  |  |  |  |  |  |  |  |
| Non-sucrose carbohydrate |  |  |  |  |  | x |  |  |  |  |  |  |  |  |  |  |  |  |  |  |
| Added sugar |  |  |  | x |  |  |  |  |  |  |  |  |  |  |  |  |  |  |  |  |
| Total fiber |  |  | ↓ |  |  |  |  |  |  | ↓ |  |  | ↑ |  |  |  |  |  |  |  |
| Water-soluble fiber |  |  | ↓ |  |  |  |  |  |  | ↓ |  |  |  |  |  |  |  |  |  |  |
| Water-insoluble fiber |  |  | ↓ |  |  |  |  |  |  | x |  |  |  |  |  |  |  |  |  |  |
| Total fat |  |  |  |  |  | x |  |  |  |  |  |  |  |  |  |  |  |  | x |  |
| Saturated fat |  |  |  |  |  | x |  |  |  |  |  |  |  |  |  |  |  |  | x |  |
| Trans fat |  |  |  |  | x |  |  | x |  |  |  |  |  |  |  | x |  |  |  |  |
| Monounsaturated fat |  |  |  |  |  | x |  |  |  |  |  |  |  |  |  |  |  |  | ↑ |  |
| Polyunsaturated fat |  |  |  |  |  | ↑ | x |  |  |  |  |  |  |  | x |  |  |  | ↑ |  |
| n-3 fatty acids | x |  |  |  |  | ↑ |  | x |  |  | x |  |  |  |  | x | x |  |  |  |
| n-6 fatty acids |  |  |  |  |  | ↑ |  | x |  |  |  |  |  |  |  | x |  |  |  |  |
| Micronutrients and minerals |  |  |  |  |  |  |  |  |  |  |  |  |  |  |  |  |  |  |  |  |
| Vitamin A |  |  |  |  |  |  |  |  |  |  |  |  |  |  |  |  |  |  | x |  |
| Vitamin C |  | x |  |  |  |  |  | x |  |  |  |  | ↑ | ↓ |  | ↑^a^ |  |  | ↓ | x |
| Vitamin D | x |  |  |  |  |  |  | x |  |  |  |  |  |  |  | ↑^a^ |  |  | x |  |
| Vitamin E |  | x |  |  |  |  | x | x |  |  |  |  |  |  | x | ↑^a^ |  |  | x | ↓ |
| Vitamin K |  |  |  |  |  |  |  |  |  |  |  |  |  |  |  |  |  |  | x |  |
| Calcium |  |  |  |  |  |  | x | x |  | x | x | x |  |  | x | x | x | ↓ | x |  |
| Iron |  |  |  |  |  |  |  |  |  |  |  |  |  |  |  |  |  |  | x |  |
| Potassium |  |  |  |  |  |  |  |  |  | ↓ |  |  |  |  |  |  |  |  | ↓ |  |
| Sodium |  |  |  |  |  |  | x |  |  |  |  |  | ↑ |  | x |  |  |  |  |  |
| Selenium |  |  |  |  |  |  |  |  |  |  |  |  |  |  |  |  |  |  | x |  |
| Magnesium |  |  |  |  |  |  | x | x | x | x |  |  |  |  | x | x |  |  | ↓ |  |
| Zinc |  |  |  |  |  |  |  |  |  |  |  |  |  |  |  |  |  |  | x |  |
| Copper |  |  |  |  |  |  |  |  |  |  |  |  |  |  |  |  |  |  | x |  |
| Folate |  |  |  |  |  |  |  | x |  |  |  |  |  |  |  | x |  |  |  |  |

HDP, hypertensive disorders of pregnancy including both pre-eclampsia and gestational hypertension; ↓, significantly lower risk with higher nutrient intake; ↑, significantly higher risk with higher nutrient intake; x, no statistically significant association.

^a^ Significantly higher risk with higher nutrient intake from diet and supplements, not from diet only.
