# Supplementary material for: The association between dietary factors and gestational hypertension and pre-eclampsia: a systematic review and meta-analysis of observational studies
Source: BMC Med. 2014 Sep 22;12:157. doi: 10.1186/s12916-014-0157-7 (PMC4192458; doi:10.1186/s12916-014-0157-7)
Supplement: Additional file 4: — Difference in unadjusted food intake between pre-eclampsia and/or gestational hypertension cases and non-cases and associations between food consumption and pre-eclampsia and/or gestational hypertension adjusted for confounding factors. [file 12916_2014_157_MOESM4_ESM.doc]

**Additional file 4** Difference in unadjusted food intake between pre-eclampsia and/or gestational hypertension cases and non-cases and associations between food consumption and pre-eclampsia and/or gestational hypertension adjusted for confounding factors

|  | Pre-eclampsia | | | | | | | | | | | | | | | | | | | | Gestational hypertension | |
| --- | --- | --- | --- | --- | --- | --- | --- | --- | --- | --- | --- | --- | --- | --- | --- | --- | --- | --- | --- | --- | --- | --- |
|  | Cohort studies | | | | | | | | | | Case–control studies | | | | | | | | | | Cohort studies | |
|  | Unadjusted | | | Adjusted | | | | | | | Unadjusted | | | | | | Adjusted | | | | Adjusted | |
|  | Borgen et al., 2012 [37] | Clausen et al., 2001 [40] | Longo-Mbenza et al., 2008 [41] | Brantsæter et al., 2011 [20] | Klemmensen et al., 2009 [26] | Triche et al., 2008 [30] | Saftlas et al., 201 0[31] | Borgen et al., 2012 [37] | Oken et al., 2007 [43] | Richardson et al., 1995 [46] | Frederick IO, 200 5[21] | Gulsen S, 2012 [23] | Atkinson JO, 1998 [53]^a^ | Reyes L, 2012 [56] | Richards DGD, 2014 [57] | Sharbaf, 2013 [59] | Frederick IO, 2005 [21] | Wei S-Q, 2009 [36] | Duvekot EJ, 2002 [54] | Zhang C, 2002 [60] | Saftlas AF, 2010 [31] | Oken E, 2007 [43] |
| Fruit and vegetables |  |  |  |  |  |  |  |  |  |  |  |  |  |  |  |  |  |  |  |  |  |  |
| Total fruit | ↓ | x |  |  | x |  |  | ↓ |  |  |  | ↓ | x | x | ↓ |  |  |  |  | x |  |  |
| Total vegetables |  | x | ↓ |  |  |  |  |  |  |  |  | ↓ | x | x | x |  |  |  |  | ↓ |  |  |
| Total fruit and vegetables |  |  |  |  |  |  |  |  |  |  | ↓ |  |  |  |  |  | ↓ |  |  | ↓ |  |  |
| Fruit juice | x |  |  |  |  |  |  |  |  |  | ↓ |  |  |  |  |  | ↓ |  |  |  |  |  |
| Bananas |  |  |  |  |  |  |  |  |  |  |  |  | x |  |  |  |  |  |  |  |  |  |
| Mangos |  |  |  |  |  |  |  |  |  |  |  |  | x |  |  |  |  |  |  |  |  |  |
| Legumes |  |  |  |  |  |  |  |  |  |  |  | ↑ |  |  | x |  |  |  |  |  |  |  |
| Grain foods |  |  |  |  |  |  |  |  |  |  |  |  |  |  |  |  |  |  |  |  |  |  |
| Cereal | x |  |  |  |  |  |  | x |  |  | x |  |  | ↑ | x |  | x |  |  |  |  |  |
| Dark bread |  |  |  |  |  |  |  |  |  |  | x |  |  |  |  |  | x |  |  |  |  |  |
| Bread |  |  |  |  |  |  |  |  |  |  |  | ↑ | x |  |  |  |  |  |  |  |  |  |
| Biscuits |  |  |  |  |  |  |  |  |  |  |  |  | x |  |  |  |  |  |  |  |  |  |
| Cake |  |  |  |  |  |  |  |  |  |  |  |  | x |  |  |  |  |  |  |  |  |  |
| Dairy |  |  |  |  |  |  |  |  |  |  |  |  |  |  |  |  |  |  |  |  |  |  |
| Total dairy |  |  |  |  |  |  |  |  |  |  |  |  | x | x |  |  |  |  |  |  |  |  |
| Low-fat dairy |  |  |  |  |  |  |  |  |  |  | x |  |  |  |  |  | x |  |  |  |  |  |
| High-fat dairy |  |  |  |  |  |  |  |  |  |  | x |  |  |  |  |  | x |  |  |  |  |  |
| Milk |  |  |  |  |  |  |  |  | x | ↑^b^ |  | ↓ | x |  | x |  |  |  | ↓ |  |  | x |
| Yogurt | x |  |  |  |  |  |  |  |  |  |  | ↓ |  |  | x |  |  |  |  |  |  |  |
| Cheese |  |  |  |  |  |  |  |  |  |  |  | ↓ | x |  | ↓ |  |  |  |  |  |  |  |
| Dairy desserts and ice cream | x |  |  |  |  |  |  |  |  |  |  | ↓ | x |  |  |  |  |  |  |  |  |  |
| Probiotic foods |  |  |  |  |  |  |  |  |  |  |  |  |  |  |  |  |  |  |  |  |  |  |
| Milk-based probiotic products |  |  |  | ↓ |  |  |  |  |  |  |  |  |  |  |  |  |  |  |  |  |  |  |
| Meat and alternatives |  |  |  |  |  |  |  |  |  |  |  |  |  |  |  |  |  |  |  |  |  |  |
| Meat |  | x | x |  |  |  |  |  |  |  |  | ↓ | ↑ | x | x |  |  |  |  |  |  |  |
| Fish |  | x |  |  |  |  |  |  | x |  |  | ↓ | x |  | x |  |  |  |  |  |  | x |
| Egg |  |  |  |  |  |  |  |  |  |  |  | ↓ | x |  |  |  |  |  |  |  |  |  |
| Chicken |  |  |  |  |  |  |  |  |  |  |  | ↓ | x |  |  |  |  |  |  |  |  |  |
| Fats |  |  |  |  |  |  |  |  |  |  |  |  |  |  |  |  |  |  |  |  |  |  |
| Total fats |  |  |  |  |  |  |  |  |  |  |  |  | x |  |  |  |  |  |  |  |  |  |
| Butter |  |  |  |  |  |  |  |  |  |  |  |  | x |  |  |  |  |  |  |  |  |  |
| Margarine |  |  |  |  |  |  |  |  |  |  |  |  | x |  |  |  |  |  |  |  |  |  |
| Confectionery |  |  |  |  |  |  |  |  |  |  |  |  |  |  |  |  |  |  |  |  |  |  |
| Sugar-containing beverages | ↑ | ↑ |  |  |  |  |  | ↑ |  |  |  |  |  |  |  |  |  |  |  |  |  |  |
| Chocolate | x |  |  |  |  | x | ↓ |  |  |  |  |  |  |  |  |  |  |  |  |  | ↓ |  |
| Caffeinated beverages |  |  |  |  |  |  |  |  |  |  |  |  |  |  |  |  |  |  |  |  |  |  |
| Tea |  |  |  |  |  |  |  |  |  |  |  |  |  |  | x | x |  | ↑^c^ |  |  |  |  |
| Coffee |  |  |  |  |  |  |  |  |  |  |  |  |  |  | x | x |  |  |  |  |  |  |
| Tea or coffee |  |  |  |  |  |  |  |  |  |  |  |  |  |  |  | x |  |  |  |  |  |  |
| Coffee and soft drinks |  |  |  |  |  |  |  |  |  |  |  |  |  |  |  |  |  | x |  |  |  |  |

↓, Significantly lower consumption for pre-eclampsia and/or gestational hypertension cases compared with non-cases (for difference in unadjusted food consumption) or significantly lower risk with higher consumption (for adjusted associations); ↑, significantly higher consumption for pre-eclampsia and/or gestational hypertension cases compared with non-cases (for difference in unadjusted food consumption) and higher risk with higher consumption (for adjusted associations); x, no statistically significant association.

^a^ Adjusted for maternal age only.

^b^ U-shaped association.

^c^ Severe pre-eclampsia (for definition, see Additional file 1: Table S3).
